# Nutritional Management of Polyendocrine Metabolic Ovarian Syndrome: Current Evidence on Dietary Strategies, Supplementation, and Lifestyle Support

**DOI:** 10.3390/nu18193218

**Published:** 2026-09-29

**Authors:** Marek Murawski, Agata Góral, Marta Maria Miszczak, Anna Prescha

**Affiliations:** 1 Clinical Department of Gynecological Surgery and Oncology, Wroclaw Medical University, 50-556 Wroclaw, Poland; marek.murawski@umw.edu.pl; 2 University Clinical Hospital, 50-556 Wroclaw, Poland; 3 Department of Dietetics and Bromatology, Wroclaw Medical University, 50-556 Wroclaw, Poland; m.miszczak@umw.edu.pl

**Keywords:** polyendocrine metabolic ovarian syndrome (PMOS), polycystic ovary syndrome (PCOS), dietary intervention, dietary patterns, dietary supplementation, gut microbiota, lifestyle intervention, metabolic health

## Abstract

Polyendocrine metabolic ovarian syndrome (PMOS), formerly known as polycystic ovary syndrome (PCOS), is a heterogeneous endocrine and metabolic disorder associated with reproductive dysfunction, hyperandrogenism, insulin resistance, chronic low-grade inflammation and an increased risk of long-term cardiometabolic disease. Lifestyle intervention remains the cornerstone of management; however, the optimal nutritional strategy continues to be debated. This narrative review summarizes current evidence on nutritional management of PMOS, including dietary approaches, the role of the gut microbiota, dietary supplementation and complementary lifestyle interventions. Current evidence indicates that no single dietary model is universally superior for all women with PMOS. Instead, different dietary approaches may improve selected anthropometric, metabolic, endocrine, and reproductive outcomes, although the consistency of these effects varies across interventions and outcomes. The most consistent benefits are observed for body weight, insulin resistance, glycolipid metabolism and cardiometabolic risk, whereas improvements in reproductive and hyperandrogenic outcomes are generally more variable. Gut microbiota modulation represents a biologically plausible mechanism linking diet with metabolic and reproductive function, although its role as a mediator of dietary effects remains insufficiently established. Adjunctive supplementation, particularly with inositols, vitamin D, omega-3 fatty acids, and selected probiotics or synbiotics, may improve selected metabolic and reproductive outcomes, although the certainty and consistency of these effects vary across supplements. Such approaches should be considered complementary to, rather than a replacement for, lifestyle management. Physical activity, behavioural support, adequate sleep and stress management further contribute to comprehensive care, although evidence is strongest for structured exercise interventions. Lifestyle approaches may also have a role in early risk modification, particularly through the prevention of excess weight gain and promotion of physical activity, although direct evidence for primary prevention of PMOS remains limited. Overall the findings support individualized and sustainable nutritional and lifestyle strategies rather than a universal therapeutic approach. Future research should focus on well-designed long-term randomized trials and the development of personalized approaches that account for clinical and metabolic heterogeneity in PMOS.

## 1. Introduction

Polyendocrine metabolic ovarian syndrome (PMOS), formerly known as polycystic ovary syndrome (PCOS), is one of the most common endocrine disorders affecting women of reproductive age worldwide [1]. The term PMOS has been proposed to better reflect the broader endocrine and metabolic nature of the syndrome, extending beyond its traditional emphasis on ovarian morphology and reproductive dysfunction. New nomenclature highlights the central role of insulin resistance, metabolic disturbances, and long-term cardiometabolic risk. Although the clinical manifestations of PMOS frequently begin during adolescence, the diagnosis is often delayed because many features of the syndrome overlap with the physiological changes in puberty, resulting in under-recognition during the early years after menarche [2,3]. Consequently, many women experience symptoms for several years before receiving a formal diagnosis in early adulthood [4]. Recent Global Burden of Disease analyses estimate that the global burden of PMOS among females aged 10–24 years increased over 1990–2021, with rises in incidence, prevalence, and disability-adjusted life years (DALYs), highlighting its growing public health relevance [5]. Therefore, greater awareness of PMOS in younger populations is important to facilitate appropriate evaluation and timely management. Because PMOS multifactorial origin complete primary prevention may not always be possible. Nevertheless, modifiable factors associated with metabolic deterioration and greater clinical severity can be targeted from an early age [2].

PMOS is a clinically heterogeneous disorder characterized by gynecologic manifestations, including menstrual irregularities, ovulatory dysfunction, infertility, and hyperandrogenism, together with a broad spectrum of metabolic abnormalities [6]. The Rotterdam criteria remain the most widely used diagnostic criteria for PMOS, and the diagnosis can be established when at least two of the following three criteria are present: (1) clinical or biochemical signs of hyperandrogenism (HA), (2) oligomenorrhea or amenorrhea (OD), and (3) polycystic ovarian morphology (PCOM) confirmed by ultrasound examination [7]. According to a recent global meta-analysis, the prevalence of PMOS among adult women is estimated at 12.1% when the Rotterdam criteria are applied, although prevalence estimates vary depending on the diagnostic criteria used [8]. Based on the Rotterdam criteria, four PMOS phenotypes have been distinguished: phenotype A (HA + OD + PCOM), phenotype B (HA + OD), phenotype C (PCOM + HA), and phenotype D (OD + PCOM). Phenotypes A and B are often called classic types and are associated with more severe symptoms, more frequent metabolic disturbances, and irregular menstrual cycles. Phenotype C (“ovulatory”) has an intermediate metabolic risk, whereas phenotype D (“nonhyperandrogenic”) is characterized by normal androgen levels and the lowest risk of metabolic disorders [9].

Insulin resistance and compensatory hyperinsulinaemia are considered central features of PMOS pathophysiology, contributing to hyperandrogenism and other endocrine abnormalities [10]. Consequently, women with PMOS are at increased risk of obesity, type 2 diabetes, dyslipidaemia, and broader cardiometabolic dysfunction, with evidence also suggesting higher rates of eating disorders [11,12]. Increasing evidence indicates that these metabolic abnormalities should be considered as integral components of the syndrome rather than merely associated comorbidities, contributing to both reproductive dysfunction and long-term cardiometabolic health consequences that may persist beyond the menopausal transition [13,14,15].

In recent years, growing recognition of the metabolic dimension of PMOS has reinforced the importance of multidisciplinary management and increased interest in non-pharmacological interventions, particularly lifestyle modification. Nutritional therapy is regarded as a core component of first-line management, not only because of its effects on body weight, but also because it can improve insulin resistance and other metabolic parameters, with potential benefits for androgen excess, menstrual function, and reproductive outcomes [16,17].

Although lifestyle intervention is universally recommended as first-line therapy, there is still no consensus regarding the optimal dietary model, macronutrient composition or the role of specific nutraceuticals in women with PMOS [18,19,20]. Therefore, a critical appraisal of current evidence is needed to identify dietary strategies supported by the strongest evidence, evaluate the role of nutraceuticals, and define priorities for future research.

## 2. Literature Search and Evidence Selection

To provide an up-to-date overview of nutritional and lifestyle management in PMOS, a comprehensive literature search was conducted in PubMed and Web of Science, including publications available up to July 2026. The search strategy combined the terms “polycystic ovary syndrome” OR “polyendocrine metabolic ovarian syndrome” with thematic keyword sets covering (i) dietary interventions and weight management (“diet”, “diet therapy”, “dietary intervention”, “nutrition”, “nutritional therapy”, “energy restriction”, “calorie restriction”, “low-energy diet”); (ii) dietary patterns and macronutrient modifications (“Mediterranean diet”, DASH, MIND, “low-glycaemic index”, “low-glycaemic load”, “low-carbohydrate diet”, “ketogenic diet”, “high-protein diet”, “time-restricted eating”, “intermittent fasting”); (iii) gut microbiota (“gut microbiota”, microbiome, dysbiosis); (iv) dietary supplementation (“dietary supplement”, supplementation, nutraceuticals, and supplement-specific terms including inositol, “vitamin D”, “omega-3 fatty acids”, probiotics, and synbiotics); (v) lifestyle and health-related behaviours (“physical activity”, exercise, “sedentary behaviour”, sleep, stress, “behavioural intervention”, yoga, smoking, tobacco, alcohol); and (vi) prevention and early risk modification (“prevention”, “risk”, adolescence, childhood, “early life”, BMI, adiposity, diet, “physical activity”, sleep). These terms were used alone or in combination with outcome-related terms, including “body weight”, “weight loss”, “body composition”, adiposity, “insulin resistance”, glucose, lipids, androgen, hormonal, menstrual, ovulation, fertility, reproductive, inflammation, and “quality of life”. Priority was given to systematic reviews, meta-analyses, randomized controlled trials, international evidence-based guidelines, and consensus statements, while high-quality observational studies and landmark publications were included when they provided important mechanistic insights or historical context. Publications not directly addressing nutritional or lifestyle interventions or potentially modifiable factors relevant to early PMOS risk, duplicate reports, and studies with limited relevance to the scope of this review were excluded from detailed analysis.

As pharmacological and surgical management was included primarily to provide clinical context for nutritional and lifestyle interventions, these sections were not subjected to the same comprehensive literature search. They were based primarily on current international evidence-based guidelines, supplemented by selected systematic reviews, meta-analyses, and original studies relevant to the therapeutic approaches discussed. Evidence concerning pharmacological and surgical treatment was summarized selectively, with emphasis on therapeutic indications, position in management, and clinically relevant considerations rather than an exhaustive review of these interventions.

## 3. Pathophysiology

The pathophysiology of PMOS is complex and remains incompletely understood. Current evidence indicates that the syndrome results from interactions between neuroendocrine, metabolic, genetic, and environmental factors, giving rise to the heterogeneous reproductive and metabolic phenotype observed in affected women [21,22,23].

One of the key mechanisms is dysregulation of the hypothalamic–pituitary–ovarian (HPO) axis, leading to an increased frequency of pulsatile gonadotropin-releasing hormone (GnRH) secretion and a predominance of luteinizing hormone (LH) over follicle-stimulating hormone (FSH). This results in excessive stimulation of ovarian theca cells, increased androgen production, and impaired follicular maturation, ultimately contributing to chronic anovulation and reproductive dysfunction [21,22].

Metabolic dysfunction represents another fundamental component of PMOS. Insulin resistance and hyperinsulinaemia are present in a substantial proportion of women with PMOS, although their severity varies across phenotypes and according to adiposity. Impaired insulin signalling contributes to reduced peripheral glucose uptake and often to compensatory hyperinsulinaemia, while elevated insulin concentrations stimulate ovarian androgen synthesis and suppress hepatic production of sex hormone-binding globulin (SHBG), thereby increasing circulating free androgen concentrations and further aggravating hyperandrogenism [10]. This bidirectional relationship between insulin resistance and androgen excess links metabolic dysfunction with reproductive abnormalities and represents one of the principal therapeutic targets of lifestyle intervention.

Hyperandrogenism plays a central role in ovarian dysfunction. Excess androgen concentrations impair follicular maturation, resulting in delayed follicular development, accumulation of small antral follicles, and chronic anovulation. Additionally, elevated anti-Müllerian hormone (AMH) concentrations, reflecting the increased number of small follicles, may further inhibit dominant follicle selection and perpetuate ovulatory dysfunction [10,22].

Beyond neuroendocrine and metabolic abnormalities, increasing evidence points to an important role of adipose tissue dysfunction and chronic low-grade inflammation in PMOS. Adipose tissue in women with PMOS exhibits altered endocrine activity, characterized by dysregulated adipokine secretion and increased production of pro-inflammatory mediators, which may further exacerbate insulin resistance and ovarian dysfunction. Although these mechanisms are incompletely understood, they are increasingly recognized as contributors to the long-term cardiometabolic complications associated with PMOS [24,25].

More recently, increasing attention has also been given to the role of the gut microbiota as well as genetic and epigenetic factors. Gut dysbiosis may influence glucose metabolism, inflammatory pathways, intestinal barrier integrity, and hormonal regulation, whereas genetic susceptibility and epigenetic modifications appear to modulate individual vulnerability to disease development and clinical expression [22,23]. Although these mechanisms have not yet translated into routine clinical practice, they provide a biological basis for emerging nutritional strategies aimed at modulating host metabolism and systemic inflammation.

## 4. Pharmacological Treatment

### 4.1. Combined Oral Contraceptive Pills

Combined Oral Contraceptive Pills (COCP) are one of the primary pharmacological treatments for PMOS. The International Evidence-Based Guidelines from 2023 recommend COCPs as first-line therapy when the therapeutic goal is regulation of menstrual cycles and reduction in clinical hyperandrogenic symptoms [26]. They should be particularly considered in women who are not planning pregnancy in the near future, allowing for both therapeutic and contraceptive benefits. However, it should be noted that COCPs have contraindications and cannot be used in all patients [27]. It is also important to consider rare but possible adverse effects, such as venous thromboembolism, myocardial infarction or stroke [28]. For women wishing to conceive, ovulation induction should be considered the primary treatment method, as will be discussed in the subsequent chapter.

### 4.2. Metformin

Metformin, a biguanide, may be used off-label in the treatment of PMOS, particularly in patients with insulin resistance, impaired glucose metabolism, and obesity. According to current International Evidence-Based Guidelines, its use is primarily considered to improve metabolic parameters and as an adjunct in the management of ovulatory dysfunction [26].

In some women, Metformin may help regulate menstrual cycles and restore ovulation, especially in those who wish to conceive and prefer not to use COCPs [29]. Metformin may be used as an addition to ovulation-inducing therapy or as monotherapy in women with PMOS who have no other factors contributing to infertility [30].

Metformin is generally well tolerated; however, it may cause gastrointestinal side effects such as nausea and diarrhea. A rare but serious complication is lactic acidosis, occurring mainly in patients with comorbid conditions [31].

### 4.3. Anti-Obesity Drugs

In recent years, drugs from the group of glucagon-like peptide-1 (GLP-1) receptor agonists have gained significant popularity. These include agents such as Liraglutide and Semaglutide. They may serve as adjunctive therapy in PMOS, particularly in obese patients in whom lifestyle interventions have failed; however, they are not considered to be a first-line treatment in PMOS [26,32].

Another anti-obesity drug is Orlistat, a gastric and pancreatic lipase inhibitor that reduces dietary fat absorption by approximately 30% [33]. Its use leads to moderate weight loss and improvement in metabolic parameters; however, its efficacy is lower compared to GLP-1 receptor agonists. Its use may be limited by frequent gastrointestinal side effects such as steatorrhea and intestinal discomfort [34]. Additionally, a 2024 meta-analysis showed that combining Orlistat with COCPs does not improve metabolic outcomes compared to COCP use alone [35].

Increasing attention is also being given to sodium-glucose co-transporter 2 (SGLT2) inhibitors, which promote urinary glucose excretion, leading to weight loss, reduced blood pressure, and improved glycemic control [36]. Results suggest improvements in metabolic parameters, further supporting their potential role in the management of metabolic disturbances [37].

### 4.4. Antiandrogens

In patients with severe symptoms of hyperandrogenism, such as hirsutism, acne, or androgenic alopecia, antiandrogen medications may be used. These include Spironolactone, Flutamide, Finasteride, and Cyproterone acetate. Currently, COCPs remain the first-line treatment for hyperandrogenism, while antiandrogens are considered to be the second-line therapy in patients with contraindications to COCPs or insufficient response to prior treatment [32,38].

It is important to note that antiandrogens can cause abnormalities in male fetal genital development [39]. Due to their teratogenic potential, they should only be prescribed to women using effective contraception.

### 4.5. Induction of Ovulation

In PMOS patients planning pregnancy, who experience anovulatory cycles, ovulation induction therapy should be applied. According to the International Evidence-Based Guidelines, first-line treatment in PMOS patients with anovulation is Letrozole, which inhibits estrogen synthesis.

The research shows that Letrozole has higher efficacy in terms of inducing ovulation and live births, while also being associated with a lower risk of multiple pregnancies, comparing to Clomiphene. Additionally, it does not cause adverse anti-estrogenic effects on the endometrium or cervical mucus, which creates better conditions for embryo implantation [30].

Clomiphene citrate is a selective estrogen receptor modulator (SERM). However, it is important to note that about 20% of patients are considered to be clomiphene-resistant [40].

The use of Metformin in combination with Clomiphene citrate is an important treatment for women with PMOS, particularly when metabolic disorders, obesity, insulin resistance, or clomiphene resistance are present [26]. Research indicates that combination therapy with Metformin and Clomiphene increases the ovulation rate compared to clomiphene monotherapy, particularly in women with clomiphene-resistant PMOS [41].

If first-line therapy (Letrozole or Clomiphene and Metformin) is ineffective, gonadotropins may be administered [26]. Due to the increased sensitivity of the ovaries to gonadotropins in women with PMOS, current guidelines emphasize the need to use low-dose regimens, particularly the “low-dose step-up” protocol, which minimizes the risk of an excessive ovarian response [42].

Despite its high efficacy, this therapy carries an increased risk of ovarian hyperstimulation syndrome (OHSS) and multiple pregnancies, which limits its use to further lines of treatment [43].

Additionally, it should be noted that although pregnancy and live birth rates after in vitro fertilization (IVF) are comparable between patients with and without PMOS, women with PMOS demonstrate a significantly lower fertilization rate and a higher risk of complications, such as miscarriage and ovarian hyperstimulation syndrome, and therefore require individualized stimulation protocols [44].

## 5. Surgical Treatment

Surgical management of PMOS is nowadays considered in patients with infertility resistant to pharmacological treatment and as an alternative to gonadotropin therapy [26]. The most commonly used method is laparoscopic ovarian drilling (LOD). It involves creating multiple small lesions on the surface of the ovary using electrical energy or a laser. This procedure leads to reduction in ovarian stroma volume and decrease in androgen production, which results in the normalization of the hypothalamic-pituitary-ovarian axis and the restoration of ovulation [45]. The research shows that LOD reduces testosterone levels and LH/FSH ratio, improves the rate of spontaneous ovulation by 30–90% and the pregnancy rate by 13–88% [46,47]. Compared to gonadotropin therapy, this method is associated with a lower risk of multiple pregnancies and OHSS [46]. However, LOD is not a risk-free procedure. The most significant complications include pelvic adhesions and the potential reduction in ovarian reserve. Thermal damage of pelvic organs, hematomas and wound infections are also possible. Factors contributing to the higher success rate of this method include a normal body mass index (BMI), elevated luteinizing hormone (LH > 10 IU/L), shorter duration of infertility, and a patient age under 35 [47]. Studies show that the higher a patient’s BMI, the lower the rate of ovulation following LOD. A similar relationship has been observed with testosterone levels [48,49].

An alternative to LOD is transvaginal ovarian drilling (TVOD) under ultrasound guidance, which is a less invasive method, because it does not require incisions in the abdominal wall. This procedure may be associated with a lower risk of adhesions developing in the smaller pelvis, which is particularly important for patients undergoing treatment for infertility. Despite the advantages of this technique, TVOD is not yet the standard of care, because, unlike laparoscopy, there is no direct view of the surgical field, which can affect the precision of the procedure and increase the risk of damaging surrounding structures [50].

The decision to proceed with surgical treatment should be made on an individual basis, taking into account the patient’s age, previous treatments, duration of infertility, body weight, and the presence of any coexisting metabolic disorders.

A summary of the main pharmacological and surgical treatment options for PMOS is presented in Table 1.

## 6. Role of Dietary Intervention and Lifestyle in PMOS Management

Lifestyle intervention, encompassing dietary modification, physical activity, and behavioral support, is widely recommended as the first-line management strategy for women with PMOS, irrespective of reproductive goals, and remains the foundation upon which adjunctive pharmacological or nutraceutical approaches are added when needed [26,57,58,59]. This rationale reflects the central role of insulin resistance, hyperinsulinaemia, excess adiposity and chronic low-grade inflammation in the pathophysiology of PMOS, all of which contribute to hyperandrogenism, ovulatory dysfunction, and long-term cardiometabolic risk [58,60,61].

Accordingly, the objectives of these interventions extend beyond weight reduction alone. Dietary and lifestyle modification can target glycaemic regulation, androgen excess, menstrual dysfunction, ovulatory disturbance, adverse body composition, and cardiometabolic risk, while individual behavioral support improves adherence and long-term sustainability [61,62,63,64]. Weight management remains especially important in women with overweight or obesity, because adiposity exacerbates insulin resistance and the clinical severity of PMOS, yet insulin resistance is also present in many lean women, supporting the relevance of lifestyle intervention across phenotypes rather than only in weight-centric care [64,65].

Within this framework, nutrition occupies a central place, but current evidence does not support a single universally optimal diet. Reviews and guideline-based syntheses instead indicate that multiple dietary patterns can improve relevant outcomes when they reduce insulin resistance, improve dietary quality and remain acceptable over time, making individualization more defensible than rigid macronutrient prescription [17,66]. This is especially relevant in a heterogeneous syndrome in which treatment priorities differ across women, including weight management, cycle regulation, fertility, hyperandrogenic symptoms, and metabolic risk reduction [6,18].

Adjunctive supplementation is best positioned within the same logic: not as a stand-alone alternative to lifestyle care, but as a potential supportive strategy for women in whom targeting insulin signalling, inflammation, oxidative stress or reproductive dysfunction is clinically relevant [19,67,68]. At the same time, broader lifestyle domains such as sleep, stress, and psychological wellbeing also influence metabolic and endocrine outcomes and may affect the feasibility and durability of dietary and exercise change, although the interventional evidence here remains less developed than for diet and physical activity [58,59,69].

### 6.1. Dietary Strategies and Approaches

#### 6.1.1. Dietary Energy Restriction

Energy restriction is one of the most consistently studied dietary strategies in women with PMOS, particularly in those with overweight or obesity [70,71]. Across randomized and interventional studies, hypocaloric diets generally improve body weight, insulin-related parameters, androgen status, and aspects of reproductive function, although the magnitude of benefit varies with intervention intensity and duration (Table 2).

Early randomized studies showed that short-term energy restriction improved body weight, insulin-related parameters, androgen status and menstrual function in women with PMOS, with broadly comparable effects across diets differing in macronutrient composition [72,73]. These findings support the view that, in many patients, the clinical benefit of diet is driven primarily by weight loss and accompanying improvements in insulin homeostasis rather than by a unique macronutrient profile [74].

Recent randomized studies further confirm these findings. In the study by Zhang et al. [75], adding dulaglutide to a calorie-restricted diet shortened the time to achieve 7% weight loss, while benefits beyond calorie restriction alone were limited to selected metabolic outcomes, suggesting that pharmacologic intensification may complement rather than replace dietary intervention as first-line treatment. Energy restriction can also be implemented within different dietary models, including ketogenic and Mediterranean [76,77], high-protein/high-fibre [78] and time-restricted eating [79] approaches, as illustrated by the studies summarized in Table 2. The effects of these dietary models in women with PMOS are discussed in greater detail in the following sections. Importantly, comparisons between such models may be confounded by differences in the magnitude of the energy deficit, making it difficult to separate the effects of dietary composition from those of restriction intensity, as illustrated by Cincione et al. [76].

**Table 2:** Effects of calorie-restricted dietary interventions on metabolic and reproductive outcomes in women with PMOS: evidence from randomized controlled trials.

Study	Design/Sample	Intervention/Comparator	Duration	Key Outcomes	Clinical Interpretation
Deshmukh et al., 2023 [70]	Open-label RCT; 40 women with obesity and PMOS	VLCD (800 kcal/day for 8 weeks, followed by stepped food reintroduction) vs. moderate energy-deficit diet (600 kcal/day deficit)	16 weeks; primary comparison at 8 weeks	Greater weight loss with VLCD at 8 weeks (−10.9% vs. −3.9%); more pronounced improvements in body composition and selected metabolic and androgen-related measures	Supports the importance of the magnitude of energy restriction for short-term weight and metabolic responses
Zhang et al., 2023 [75]	Open-label RCT; 68 women with overweight/obesity and PMOS	CRD vs. dulaglutide + CRD	Until 7% weight loss; maximum 6 months	Dulaglutide + CRD achieved target weight loss faster and produced greater reductions in HbA1c and postprandial glucose; changes in VAT, most other metabolic measures, hormones and menstrual frequency did not differ significantly	Suggests that pharmacological intensification may accelerate diet-induced weight loss without consistently enhancing broader benefits at comparable weight-loss targets
Pandurevic et al., 2023 [77]	Open-label RCT; 30 women with obesity and PMOS	VLCKD followed by LCD vs. Mediterranean LCD	16 weeks	Greater reductions in body weight/BMI, fat mass and free testosterone and higher ovulation rate with the VLCKD-based intervention	Suggests that the dietary strategy used within an energy-restricted intervention may influence selected metabolic and reproductive outcomes
Cincione et al., 2022 [76]	RCT; 144 women with overweight/obesity and PMOS completing treatment	Very-low-calorie ketogenic diet vs. moderately hypocaloric Mediterranean diet	45 days	Both interventions improved anthropometric and biochemical parameters, with larger changes reported in the ketogenic group	Interpretation is limited by simultaneous differences in energy restriction and dietary composition, preventing their independent effects from being distinguished
Dou et al., 2024 [78]	RCT; 90 women with overweight/obesity and PMOS	CRD vs. high-protein diet vs. high-protein/high-fibre diet	8 weeks	All diets produced >5% weight loss and improved HOMA-IR and oxidative-stress measures; high-protein diets better preserved lean mass, with the greatest VFA reduction in the high-protein/high-fibre group	Suggests that macronutrient composition may modify body-composition and oxidative-stress responses within weight-loss interventions
Zheng et al., 2025 [79]	RCT; 93 women with overweight/obesity and PMOS	Calorie-restricted diet (1200–1500 kcal/day) with an 8 h eating window vs. the same calorie-restricted diet without time restriction	16 weeks	Greater weight loss with the addition of time-restricted eating (−7.47 vs. −4.38 kg); insulin resistance improved in both groups	Adding a restricted eating window to calorie restriction may enhance weight loss, suggesting that meal timing may modify the response to energy-restricted dietary interventions

BMI, body mass index; CRD, calorie-restricted diet; HbA1c, glycated haemoglobin; HOMA-IR, homeostasis model assessment of insulin resistance; LCD, low-calorie diet; PMOS, polyendocrine metabolic ovarian syndrome; RCT, randomized controlled trial; VAT, visceral adipose tissue; VLCD, very-low-calorie diet; VFA, visceral fat area; VLCKD, very-low-calorie ketogenic diet.

Meta-analytic evidence supports energy restriction as an effective strategy for women with PMOS and overweight or obesity, with the most consistent benefits observed for body weight, body composition and insulin-related outcomes. A recent meta-analysis restricted to randomized controlled trials in women with BMI ≥ 25 kg/m^2^ found improvements in weight-related, glycaemic, hormonal, and fertility-related outcomes, with subgroup analyses also supporting calorie-restricted dietary approaches [80]. Network meta-analysis ranked low-calorie diets highly for BMI reduction and low-calorie diet combined with metformin for weight loss, whereas the most favourable rankings for insulin-related outcomes were observed with Dietary Approaches to Stop Hypertension (DASH); conventional meta-analysis likewise showed improvements in insulin resistance and anthropometric outcomes across several dietary interventions rather than specifically with calorie restriction [17,81]. A broader recent synthesis of weight-loss strategies in women with overweight or obesity and PMOS likewise identified calorie-restricted dietary approaches among the potentially effective options, while emphasizing that metabolic, hormonal, and reproductive responses vary across interventions [82]. In pooled before–after analyses, diet-induced weight loss was also associated with improvements in inflammatory and selected androgen-related markers, although effects on individual hormonal outcomes are less consistent [83]. Broader lifestyle syntheses similarly indicate improvements in free androgen index (FAI) and SHBG, particularly when diet and exercise are combined [84,85].

Reproductive outcomes are more heterogeneous. Dietary interventions have been associated with improvements in clinical pregnancy, ovulation and menstrual regularity; however, subgroup analyses do not indicate that these benefits are consistently attributable to calorie restriction itself [71]. In particular, hypocaloric diets did not improve ovulation rates in subgroup analyses, while improvements in clinical pregnancy and menstrual regularity were more evident in interventions without calorie restriction [71]. Thus, the reproductive benefits of dietary intervention should not be interpreted as a direct consequence of weight loss alone.

Intervention duration and the magnitude of weight loss may further influence treatment response. Longer dietary interventions have been associated with greater reproductive benefits [71], while weight-loss interventions produced greater increases in SHBG and reductions in triglycerides than weight-maintenance approaches [84]. Evidence for pregnancy-related outcomes remains less certain. A Cochrane review found insufficient data on live birth and miscarriage, while a more recent systematic review reported only low-certainty evidence for improved pregnancy rates following dietary interventions [84,86].

Energy restriction therefore represents a well-supported strategy when weight loss is clinically indicated. Its relevance may differ according to the predominant therapeutic goal: subgroup analyses suggest more consistent benefits for androgen-related outcomes, whereas reproductive benefits, including ovulation and menstrual outcomes, do not appear to depend consistently on energy restriction. This distinction should be considered alongside the patient’s clinical and metabolic characteristics when selecting a dietary strategy.

#### 6.1.2. Carbohydrate Restriction and Glycaemic Approaches

Carbohydrate-focused dietary strategies in women with PMOS primarily target three interconnected pathophysiological features of the disorder, namely dysregulated glucose metabolism, insulin resistance, and hyperandrogenism. Overall, evidence from clinical studies and meta-analyses indicates that low-carbohydrate, ketogenic, and low-glycaemic index (low-GI) dietary approaches improve insulin-related outcomes, whereas their effects on androgen excess are generally favourable but less consistent across dietary models (Table 3) [71,87,88,89].

Among these approaches, low-carbohydrate and ketogenic diets have shown short-term improvements in fasting insulin, insulin resistance, body weight and other obesity-related metabolic outcomes, particularly in women with overweight or obesity, although comparative evidence does not establish their overall superiority over other dietary strategies [90,91]. Individual randomized trials comparing lower-carbohydrate or ketogenic approaches with low-fat, Mediterranean or moderate-carbohydrate diets generally report favourable effects on body weight, insulin-related measures and androgen-related outcomes, although the magnitude and pattern of benefit vary between dietary models (Table 3) [89,92,93,94]. Importantly, favourable metabolic and androgen-related effects of carbohydrate modification have been observed both in the absence of intentional energy restriction [92] and during energy restriction, where differences between dietary models were evident despite comparable energy intake [94].

These findings from individual randomized trials are supported by a meta-analysis of eight RCTs, which demonstrated reductions in BMI and homeostasis model assessment of insulin resistance (HOMA-IR) and improvements in selected lipid outcomes with low-carbohydrate diets compared with control diets. Total testosterone was not significantly reduced overall, although longer interventions were associated with more favourable androgen-related outcomes [87].

**Table 3:** Characteristics and main findings of studies evaluating carbohydrate-focused dietary interventions in women with PMOS.

Study	Design/Sample	Intervention/Comparator	Duration	Key Outcomes	Clinical Interpretation
Paoli et al., 2020 [89]	Single-arm intervention; 14 women with overweight and PMOS	Ketogenic Mediterranean diet	12 weeks	Reduced body weight and visceral adiposity, HOMA-IR and triglycerides; testosterone and DHEAS decreased and SHBG increased	Suggests concurrent metabolic and androgen-related responses to ketogenic carbohydrate restriction; interpretation is limited by the absence of a control group
Gower et al., 2013 [92]	Randomized crossover trial; 30 women with PMOS	Eucaloric lower-carbohydrate diet (41% carbohydrate) vs. eucaloric standard diet (55% carbohydrate)	8 weeks each, with 4-week washout	Lower-carbohydrate diet reduced fasting insulin, fasting glucose, HOMA-IR and total testosterone and increased insulin sensitivity	Supports metabolic and androgen-related effects of moderate carbohydrate reduction in the absence of intentional energy restriction
Sharifi et al., 2024 [93]	RCT; 46 women with overweight/obesity and PMOS	Ketogenic diet vs. portfolio moderate-carbohydrate diet	8 weeks	Both diets improved anthropometric, glycaemic and hormonal outcomes; ketogenic diet produced greater reductions in BMI and LH, whereas the portfolio diet produced greater reductions in LDL-C and triglycerides	Different dietary approaches produced distinct profiles of metabolic and hormonal response
Mei et al., 2022 [94]	RCT; 72 women with overweight and PMOS	Energy-restricted Mediterranean diet with low carbohydrate content vs. energy-restricted low-fat diet	12 weeks	Greater reductions in body weight/adiposity, HOMA-IR, testosterone and atherogenic lipids with the Mediterranean/low-carbohydrate diet; menstrual recovery was frequent in both groups, with no significant between-group difference	Supports benefits of lower carbohydrate intake for selected metabolic and androgen-related outcomes under comparable energy restriction
Marsh et al., 2010 [95]	RCT; 96 premenopausal women with overweight/obesity and PMOS	Ad libitum low-GI diet vs. macronutrient-matched conventional healthy diet	12 months or until 7% weight loss	Greater improvement in insulin sensitivity and menstrual cyclicity with low-GI diet (95% vs. 63%); significant diet–metformin interaction for insulin sensitivity	Provides longer-term evidence for low-GI dietary management, although high attrition (49%) limits interpretation
Sordia-Hernandez et al., 2016 [96]	RCT; 37 women with PMOS and anovulation	Low-GI diet vs. normal-GI diet	3 months	Higher proportion of ovulatory cycles with low-GI diet (24.6% vs. 7.4%)	Provides direct evidence for a reproductive outcome in women with PMOS and anovulation

BMI, body mass index; DHEAS, dehydroepiandrosterone sulfate; HOMA-IR, homeostasis model assessment of insulin resistance; LDL-C, low-density lipoprotein cholesterol; LH, luteinizing hormone; low-GI, low-glycaemic-index; PMOS, polyendocrine metabolic ovarian syndrome; RCT, randomized controlled trial; SHBG, sex hormone-binding globulin.

Low-GI dietary approaches have also been investigated as a strategy to improve insulin sensitivity in women with PMOS (Table 3). Intervention studies indicate improvements in insulin-related and androgen-related outcomes as well as menstrual regularity, although some of these effects appear to be associated with concurrent weight loss and improvements in insulin resistance [97]. In women with PMOS and anovulation, a small randomized trial also reported a higher proportion of ovulatory cycles with a low-GI than with a normal-GI diet, providing some evidence for a potential reproductive benefit of this approach [96]. Evidence for low-glycaemic load (GL) diets remains limited and less consistent because relatively few randomized controlled trials have been available for pooled analyses [98].

A meta-analysis of ten RCTs demonstrated that low-GI diets improved insulin-related measures, lipid profile, waist circumference and total testosterone compared with high-GI diets, while effects on fasting glucose, body weight and the free androgen index were not significant [88]. Importantly, subgroup analysis indicated that the reduction in HOMA-IR was evident in energy-restricted trials, suggesting that carbohydrate quality and energy restriction may have contributed jointly to this effect.

Subgroup analyses further suggested that low-carbohydrate dietary interventions were associated with the most favourable reproductive outcomes, whereas calorie restriction appeared particularly important for improving hyperandrogenism [71]. More recent evidence further suggests that high-fibre and low-GI dietary patterns improve HOMA-IR, selected lipid outcomes, and androgen-related measures including SHBG and FAI, while emphasizing substantial heterogeneity between studies and the limited evidence base regarding low-GL interventions [99].

Meta-analytic evidence on ketogenic diets requires cautious interpretation. A recent systematic review and meta-analysis found greater reductions in BMI, body weight, waist circumference, HOMA-IR and LH with ketogenic diets compared with other dietary interventions, whereas effects on androgen and lipid parameters were inconsistent. However, substantial heterogeneity, variation in study quality and the inclusion of observational studies limit the certainty of these findings [100].

Collectively, the available evidence suggests that carbohydrate-focused dietary strategies differ in the profile of outcomes they most consistently influence. Such differences may be clinically relevant when individualizing dietary management according to the predominant therapeutic goal, including metabolic dysfunction, hyperandrogenism, or reproductive disturbances.

#### 6.1.3. Protein-Enriched Dietary Strategies

Beyond carbohydrate restriction, several studies have investigated whether modifying dietary macronutrient composition, particularly by increasing protein intake, provides additional benefits in women with PMOS. The rationale for these approaches is that a higher protein intake may enhance satiety, preserve lean body mass during weight loss, and improve insulin sensitivity, thereby augmenting the metabolic effects of energy restriction.

Evidence from randomized trials suggests that the benefits of higher-protein diets are selective rather than universal (Table 4). In overweight or obese women with PMOS, a hypocaloric high-protein, low-GL diet produced greater reductions in insulin-related and inflammatory markers than a conventional hypocaloric diet despite similar weight loss, while both interventions similarly reduced testosterone concentrations [98]. By contrast, another randomized study comparing high-protein and normal-protein diets found comparable reductions in body weight and composition and testosterone, with no clear differences in metabolic markers between dietary approaches, indicating no clear additional short-term benefit of increasing protein content [101].

These findings are supported by the 2024 systematic review and meta-analysis, which demonstrated that high-protein diets modestly reduced fasting insulin and HOMA-IR compared with isocaloric balanced diets [102]. However, no significant advantages were observed for body weight, abdominal adiposity, lipid profile or reproductive hormones, suggesting that increasing dietary protein does not confer broad superiority over balanced diets with comparable energy intake. Substantial heterogeneity was observed for HOMA-IR, and the pooled effect was sensitive to individual studies, indicating variability in the metabolic response across studies.

Current evidence indicates that modifying macronutrient composition beyond calorie restriction may refine metabolic responses in PMOS, with the most consistent benefit observed for insulin-related outcomes. In contrast, effects on body weight, lipid metabolism, and reproductive outcomes appear broadly comparable to those achieved with balanced hypocaloric diets. Thus, although higher-protein dietary strategies represent a reasonable option for individualized nutritional management, the available evidence does not support their superiority over other energy-restricted dietary approaches.

#### 6.1.4. Dietary Patterns and Combined Dietary Models in PMOS

Evidence from interventional, observational, cross-sectional, and meta-analytic studies supports a role for dietary patterns in PMOS, although the strength and nature of the evidence vary across dietary approaches [14,17,71,82]. Randomized trials have evaluated a range of whole-diet patterns and more targeted dietary approaches, including DASH, Mediterranean, high-fibre, and diets low in advanced glycation end products (AGEs), as well as the Mediterranean-DASH Intervention for Neurodegenerative Delay (MIND) diet; selected RCTs are summarized in Table 5. Observational and cross-sectional studies provide complementary evidence on associations between habitual dietary patterns, diet quality, and clinical or metabolic characteristics of PMOS.

##### Interventional Studies

Findings from individual RCTs and comparative meta-analytic evidence identify DASH as one of the more promising whole-diet patterns in PMOS. An energy-restricted DASH diet produced greater improvements in body composition and selected androgen-related and antioxidant outcomes than an energy-restricted control diet [103]. In network meta-analysis, DASH ranked highest for HOMA-IR, fasting glucose, fasting insulin and triglycerides, although these rankings should be interpreted cautiously because direct comparisons between dietary patterns were limited [17,81].

Mediterranean-style low-carbohydrate interventions also appear promising; however, they represent hybrid dietary models rather than evaluations of the Mediterranean diet alone. Their metabolic and reproductive benefits are discussed in the previous section on carbohydrate restriction [94]. In a recent meta-analysis of randomized controlled trials in women with PMOS and BMI ≥ 25 kg/m^2^, subgroup analysis suggested a marked reduction in LH with Mediterranean dietary interventions, although this estimate was based on a single trial [80]. A 12-week pilot RCT of an ad libitum Mediterranean diet found the intervention feasible and acceptable, with significantly higher adherence than healthy-eating advice, but it was designed primarily for feasibility rather than efficacy testing [104].

The MIND diet represents another whole-diet model, combining Mediterranean and DASH principles. A recent RCT primarily showed improvements in depression, anxiety, and selected PMOS quality-of-life domains, without broad short-term effects on metabolic or hormonal outcomes [105].

More mechanistic but narrower support for efficacy of dietary patterns in PMOS pro-vide low-AGE and high-fiber strategies. Notably, a low-AGE diet has been studied specifically in women with phenotype A PMOS and overweight. In a 12-week RCT, low-AGE and standard weight-loss diets produced similar weight loss, but the low-AGE diet produced greater improvements in glycaemic, lipid, inflammatory, and androgen-related outcomes [106]. The restriction of the study population to phenotype A should be considered when interpreting the generalizability of these findings. A high-fibre diet improved metabolic, inflammatory, reproductive, and brain–gut peptide outcomes in both groups, but the acarbose-combination arm improved hyperinsulinemia and insulin resistance more, limiting conclusions regarding the independent dietary effect [107].

Meal timing represents another emerging dimension of dietary intervention in PMOS. Time-restricted eating, the most extensively studied form of intermittent fasting in women with PMOS, has shown promising effects on body weight, insulin resistance, selected markers of hyperandrogenism, and menstrual regularity [108,109]. A broader meta-analysis of intermittent fasting interventions similarly reported favourable effects on body weight, insulin-related outcomes, and selected androgen-related measures, although the evidence remained preliminary [110]. A more recent meta-analysis focusing specifically on time-restricted eating found significant improvements in insulin sensitivity, including HOMA-IR and quantitative insulin sensitivity check index (QUICKI), whereas evidence for broader metabolic, anthropometric, and hormonal benefits was less consistent [111]. Subsequently, a 6-month randomized controlled trial found that time-restricted eating without calorie tracking and conventional daily calorie restriction produced similar reductions in body weight, with both interventions resulting in greater weight loss than no dietary intervention [112]. However, the evidence base remains limited, and heterogeneity in intervention protocols and comparators makes it difficult to determine whether the observed benefits are attributable specifically to time restriction or to accompanying changes in energy intake and body weight [110,111,113,114].

##### Observational and Cross-Sectional Evidence

Observational evidence suggests that diet quality and habitual dietary patterns are associated with PMOS and some of its clinical and metabolic characteristics, although findings are not entirely consistent across studies. In a 240-person case–control study, a quasi-Western dietary pattern was positively associated with PMOS, whereas a healthy diet was inversely associated with PMOS after adjustment [115]. In the same study, women with PMOS consumed more sugars, sweet desserts, soft drinks, processed meat, red meat, salt, and tea/coffee than controls. More recently, a case–control study of 367 women found that greater adherence to the MIND diet, which integrates elements of the Mediterranean and DASH dietary patterns, was associated with substantially lower odds of PMOS. Women in the highest adherence quartile had approximately 84% lower adjusted odds of PMOS than those in the lowest quartile (adjusted odds ratio [AOR] = 0.166, 95% CI 0.073–0.378). Higher MIND diet adherence was also associated with lower odds of phenotypes A, C, and D, but not phenotype B [116].

Cross-sectional studies provide additional clinical and metabolic detail but cannot establish causality. In 200 women with PMOS, higher Dietary Inflammatory Index (DII) scores were associated with higher fasting glucose, high-sensitivity C-reactive protein (hs-CRP), erythrocyte sedimentation rate (ESR), prolactin, FSH and LH after adjustment, while lipid associations attenuated and no independent association remained for total testosterone [117]. In a larger retrospective cross-sectional study of 420 women with PMOS, greater Mediterranean diet adherence was weakly associated with lower BMI, body fat percentage, C-reactive protein (CRP) and 1 h glucose levels during an oral glucose tolerance test (OGTT), although associations with other glycaemic markers were inconsistent [118].

Overall, current evidence supports potential benefits of whole-diet and targeted dietary approaches in PMOS, although the evidence base varies considerably across individual strategies. Metabolic outcomes have been evaluated most consistently across dietary interventions, whereas reproductive and hormonal responses appear more dependent on the specific intervention and outcome assessed. Additional long-term randomized controlled trials are needed to determine the comparative effectiveness of specific dietary approaches.

#### 6.1.5. Summary of Evidence from Meta-Analyses and Systematic Reviews

The systematic reviews and meta-analyses summarized in Table 6 do not identify a single dietary strategy as consistently superior across anthropometric, metabolic, endocrine, and reproductive outcomes. Energy-restricted approaches are particularly relevant when weight loss is clinically indicated, with evidence supporting reductions in body weight and adiposity and improvements in selected metabolic and androgen-related outcomes. However, comparative analyses indicate that metabolic benefits are not specific to energy restriction, while improvements in reproductive outcomes have also been observed across heterogeneous dietary interventions [17,71,80,81].

Carbohydrate-focused approaches show a somewhat different profile. Low-carbohydrate and low-GI dietary interventions have demonstrated improvements in insulin resistance and selected lipid and androgen-related outcomes, although effect sizes and heterogeneity vary substantially across analyses [87,88,99]. Evidence for ketogenic diets also suggests short-term anthropometric and metabolic benefits, but interpretation is limited by mixed study designs, substantial heterogeneity for several outcomes, and limited long-term evidence [100]. By contrast, higher-protein diets appear to provide a narrower additional benefit, primarily for insulin-related outcomes, without consistent superiority for body weight, lipid profile, or reproductive hormones [102]. Recent evidence on time-restricted eating suggests improvements in selected measures of insulin sensitivity and high-density lipoprotein cholesterol (HDL-C), although the evidence is currently based on only four RCTs with modest sample sizes and heterogeneous intervention protocols [111].

Reproductive outcomes further illustrate the heterogeneity of dietary response. Across 20 RCTs, dietary interventions were associated with higher clinical pregnancy and ovulation rates, although individual reproductive outcomes were often based on relatively few trials and responses varied across dietary approaches [71].

Taken together, the available evidence supports tailoring dietary strategies to the predominant clinical and metabolic characteristics, therapeutic goals, and need for weight management rather than prescribing a single optimal diet for all women with PMOS. Direct comparisons remain constrained by heterogeneity in diagnostic criteria, participant characteristics, dietary protocols, intervention duration, and outcome selection, as well as the predominantly short duration and small sample size of many trials. These limitations currently preclude firm conclusions regarding the superiority of specific dietary strategies or the development of phenotype-specific dietary recommendations.

### 6.2. Gut Microbiota as a Putative Mediator of Dietary Effects in PMOS

Emerging evidence suggests that the gut microbiota may represent one mechanistic link between diet, metabolic dysfunction and reproductive abnormalities in PMOS. Human observational studies consistently report alterations in gut microbial composition in women with PMOS, although the specific taxa differ across cohorts and methodologies [119,120,121]. Meta-analytic and systematic review data indicate reduced microbial diversity in many PMOS populations and a pattern characterized by depletion of short-chain fatty acid-producing and bile-acid-metabolizing bacteria, together with enrichment of taxa associated with more pro-inflammatory microbial profiles [121,122,123].

These findings are relevant to dietary research because gut microbial alterations in PMOS have been linked to insulin resistance, androgen excess, obesity-related traits, inflammatory signaling and gut–brain mediators, all of which are potential targets of lifestyle intervention [123,124,125]. Mechanistic work further supports biological plausibility, including evidence for a gut microbiota–bile acid–interleukin-22 axis that may influence ovarian function, insulin resistance and infertility in PMOS [126]. Microbiota-derived metabolites, including short-chain fatty acids (SCFAs), indoles and bile acids, may therefore provide potential signaling pathways linking changes in the intestinal environment with metabolic and reproductive function [127].

Interventional evidence in women with PMOS has focused predominantly on microbiota-targeted supplementation rather than on whether whole-diet interventions modify clinical outcomes through changes in the gut microbiota. Systematic reviews of randomized trials suggest that probiotics, prebiotics, synbiotics and other microbiota-targeted interventions may improve selected metabolic and hormonal outcomes [127,128]. These findings support the potential clinical relevance of microbiota modulation but do not establish that the gut microbiota mediates the effects of dietary interventions.

Thus, although microbiota modulation remains a biologically plausible mechanism linking diet with metabolic and reproductive function in PMOS, direct evidence that specific dietary interventions exert their clinical effects through reproducible changes in gut microbial composition or function remains limited [120,121,123].

### 6.3. Efficacy of Dietary Supplements in PMOS

The dietary supplement literature in PCOS suggests potential adjunctive benefits, especially for insulin resistance, selected lipid outcomes, hyperandrogenism and some reproductive endpoints, but effects differ by supplement class and the certainty is often limited by heterogeneity, short follow-up, and variable dosing [19,129,130]. Among the wide range of supplements investigated in PCOS, inositols, vitamin D, omega-3 fatty acids and N-Acetylcysteine (NAC) have been evaluated across multiple intervention studies and evidence syntheses and are therefore considered in greater detail below. Selected intervention studies illustrating different clinical contexts and reported effects are summarized in Table 7, whereas Table 8 presents evidence from systematic reviews and meta-analyses, providing a broader assessment of the consistency and certainty of these effects. Other supplements, for which the evidence base is more limited or less developed, are considered subsequently.

#### 6.3.1. Inositol

Inositol is among the most extensively studied supplements in PCOS, with evidence suggesting improvements in selected metabolic and reproductive outcomes. A meta-analysis of 26 RCTs reported improvements in menstrual-cycle regularity and selected anthropometric, metabolic and androgen-related outcomes [148]. A guideline-linked review was more cautious, concluding that inositol appears to benefit some metabolic measures and D-chiro-inositol may improve ovulation, but overall evidence remains limited and inconclusive [141].

Newer umbrella evidence sharpens that picture rather than overturning it. An umbrella review of 13 meta-analyses found no high-quality evidence, but reported moderate-quality support for improvements in total testosterone, free testosterone, SHBG, HOMA-IR, pregnancy and ovulation, with myo-inositol or myo-inositol plus folate generally performing better than D-chiro-inositol alone [149]. Accordingly, current evidence supports possible benefits of inositol for selected metabolic and reproductive outcomes, but does not establish a specific formulation or dose as clinically superior [141,149,150,151].

#### 6.3.2. Vitamin D

Vitamin D supplementation has been associated with improvements in selected metabolic, inflammatory, androgenic and reproductive markers, although effects are not consistent across outcomes or supplementation regimens [143,144].

The inflammatory signal is one of the more reproducible findings. A dedicated meta-analysis of seven RCTs showed that vitamin D reduced hs-CRP and malondialdehyde (MDA) and increased total antioxidant capacity (TAC), without clear effects on nitric oxide or glutathione [152]. Another review focused on insulin resistance found that vitamin D worked best as a co-supplement or at continuous doses below 4000 IU/day for lowering fasting glucose and HOMA-IR, which helps explain why pooled effects vary across trials using different regimens [153].

#### 6.3.3. Omega-3 Fatty Acids and N-Acetylcysteine (NAC)

Current evidence suggests that omega-3 fatty acids improve insulin resistance, triglycerides and both inflammatory and oxidative stress markers in women with PMOS, although the overall strength of evidence remains moderate. The umbrella review found moderate-certainty evidence that fish oil lowers fasting insulin and HOMA-IR and that omega-3 fatty acids lower triglycerides [129]. A recent network meta-analysis ranked omega-3 fatty acids as superior for lowering HOMA-IR, while another ranked them highly for fasting glucose reduction [154,155]. These comparative findings complement conventional meta-analytic evidence indicating effects on selected inflammatory, oxidative-stress and androgen-related outcomes [145].

The overall pattern of evidence indicates that omega-3 supplementation exerts stronger metabolic and anti-inflammatory effects than effects on body weight or body composition. Clinical-trial synthesis reported reductions in insulin resistance, total cholesterol and triglyceride levels and some androgen-related measures, while anthropometric effects were usually small or inconsistent [156]. For NAC, evidence spans both metabolic applications and its use as an adjunct to ovulation induction. Pooled analyses suggest potential reproductive benefits, including ovulation, pregnancy and live birth outcomes, whereas metabolic effects appear more selective [146,147].

#### 6.3.4. Other Supplements

Evidence syntheses have also evaluated a broader range of nutritional supplements, suggesting potential benefits of chromium for fasting insulin and selected oxidative stress markers, coenzyme Q10 for lipid metabolism, and curcumin for fasting glucose and insulin resistance [67,151]. Microbiota-targeted supplementation, including probiotics and synbiotics, has also shown favourable effects on selected insulin-related and hormonal outcomes, although the certainty of evidence varies across outcomes [127]. These interventions and their potential relevance to the gut microbiota are discussed in greater detail in Section 6.2. Vitamin E alone or combined with omega-3 fatty acids or magnesium improved selected lipid parameters, hs-CRP, hirsutism score and nitric oxide, but did not clearly improve glycemic indices, anthropometrics or most hormonal outcomes [157].

Berberine supplementation in PMOS remains promising but underdeveloped. Network evidence comparing oral insulin sensitizers that berberine may lower triglycerides more effectively than metformin, while the combination of metformin and berberine may reduce total testosterone and BMI more than metformin alone, though these findings were based on only a few low-quality trials [150]. One 90-day multicenter trial of berberine phytosome (enhanced-bioavailability formulation) reported more regular menstruation, more normal ovarian morphology and some improvements in acne and hirsutism, without clear between-group metabolic or hormonal differences at study end [157].

### 6.4. Lifestyle Approaches Beyond Nutrition

Lifestyle management in PMOS extends beyond dietary modification and includes physical activity, reduction in sedentary behaviour, optimization of sleep, stress management and behavioural intervention, together with other health-related behaviours such as smoking and alcohol consumption The strength and type of evidence differ substantially across these lifestyle domains. Physical activity and structured exercise have been evaluated extensively in both observational and interventional studies, whereas other lifestyle components have been less extensively investigated and, in some domains, research remains predominantly observational [58,158,159].

Observational studies indicate that women with PMOS tend to engage in less physical activity and more sedentary behaviour than women without the syndrome. In a population-based study including 7466 women, lower physical activity was independently associated with higher BMI, while women with PMOS also reported greater daily sitting time [65]. Sedentary behaviour exceeding seven hours per day was associated with poorer PMOS-specific quality of life independently of physical activity, anxiety and depression [160]. More recent analyses extend these observations across different age groups and populations, showing lower levels of vigorous physical activity and greater sedentary or screen time among women with PMOS [161,162]. These findings are supported by a systematic review and meta-analysis of observational studies showing lower total physical activity in women with PMOS compared with controls [163]. Although these associations cannot establish causality, they identify physical inactivity and prolonged sedentary behaviour as potentially modifiable components of the broader lifestyle profile accompanying PMOS [11]. Selected studies addressing non-dietary lifestyle factors are summarized in Table 9.

Evidence from structured exercise interventions is considerably stronger than that available for other non-dietary lifestyle approaches (Table 9). Systematic reviews and meta-analyses consistently demonstrate improvements in cardiorespiratory fitness and measures of central adiposity, with additional benefits for selected metabolic outcomes [159,160,169] (Table 10). The magnitude and consistency of metabolic effects vary according to the outcome and exercise modality, however. Aerobic exercise has been associated with improvements in fasting insulin and selected lipid parameters, whereas evidence for resistance training remains more limited [169]. Effects on androgen-related outcomes are also less consistent: pooled analyses have not demonstrated clear overall effects on total testosterone, dehydroepiandrosterone sulfate (DHEA-S), or SHBG, although reductions in testosterone have been observed in analyses restricted to aerobic exercise [170]. Thus, the current evidence supports exercise primarily for its established benefits for physical fitness, body composition and selected cardiometabolic outcomes, while its endocrine effects require more cautious interpretation.

The practical implementation of physical activity may also be important. In a randomized feasibility trial, a lifestyle physical activity intervention designed to reduce sedentary behaviour achieved substantially greater adherence than a supervised aerobic exercise programme, although both intervention approaches were associated with favourable changes in selected outcomes [164]. As this study was designed primarily to assess feasibility rather than efficacy, these findings should not be interpreted as demonstrating superiority of lifestyle physical activity over structured exercise. Nevertheless, they highlight adherence and feasibility as relevant considerations when translating exercise recommendations into long-term lifestyle management.

Yoga represents a distinct mind–body approach that combines physical activity with breathing, relaxation and stress-management techniques. In an active-controlled randomized trial in adolescent girls with PMOS, a 12-week holistic yoga programme produced greater improvements in insulin resistance and selected endocrine and menstrual outcomes than conventional physical exercise [167,168]. These findings are supported by a systematic review and meta-analysis reporting improvements in selected metabolic, endocrine and menstrual outcomes, although the certainty of evidence was low [172]. A more recent systematic review of randomized trials also identified potential anthropometric, metabolic, endocrine, menstrual and psychological benefits of yoga-based interventions, while meditation and mindfulness-based approaches showed potential benefits primarily for psychological outcomes; however, small samples, heterogeneous protocols and diverse outcome measures continue to limit firm conclusions [174].

Behavioural and psychological interventions represent a less extensively studied component of PMOS management. The available randomized evidence is limited and heterogeneous, with behavioural strategies often insufficiently described [58]. Nevertheless, interventions incorporating behavioural modification or cognitive behavioural therapy have reported improvements in quality of life, depressive and anxiety symptoms, self-esteem, and perceived general health, with some studies also reporting favourable changes in body weight or selected metabolic outcomes [58,173]. Current evidence therefore supports a potential role for these approaches particularly in addressing psychological wellbeing and facilitating sustained lifestyle change, while their independent effects on metabolic and reproductive outcomes remain uncertain.

Eating disorders and disordered eating represent an additional consideration in lifestyle management in PMOS. Evidence from systematic reviews, including a recent meta-analysis, indicates an increased risk of eating disorders and disordered eating in women with PMOS, particularly binge-eating disorder and bulimia nervosa [12,175]. This is particularly relevant when implementing dietary and weight-management interventions, as eating disorders and disordered eating should be considered regardless of body weight and may require modification of the therapeutic approach [26]. Assessment of eating behaviour and psychological wellbeing may therefore be important when individualizing lifestyle interventions, with appropriate multidisciplinary support when an eating disorder or disordered eating is identified.

Sleep represents another clinically relevant but comparatively underinvestigated lifestyle domain. Women with PMOS report a greater burden of insomnia, abnormal sleep patterns, and sleep-apnoea-related risk, alongside higher levels of psychological stress [11,162]. Recent observational evidence further suggests that sleep quality may be closely related to the broader metabolic and lifestyle profile of PMOS. In a case–control study, poorer sleep quality in women with PMOS was associated with greater adiposity, less favourable metabolic and inflammatory characteristics, and lower adherence to the Mediterranean diet; in multivariable analyses, Mediterranean diet adherence emerged as the strongest overall predictor of sleep quality [166]. These findings reinforce the interrelationship between sleep, adiposity, and lifestyle quality but do not establish the direction or causality of these associations. Importantly, intervention studies specifically targeting sleep or stress in women with PMOS remain scarce, and evidence is currently insufficient to determine whether improving these behaviours independently modifies metabolic or reproductive outcomes [58].

Smoking and alcohol consumption represent additional health-related behaviours relevant to PMOS management. The 2023 International Evidence-based Guideline recommends consideration and optimization of both behaviours as part of preconception care in women with PMOS [26]. PMOS-specific research is more extensive for tobacco exposure than for alcohol consumption. A recent meta-analysis of nine observational studies involving 3053 women with PMOS associated active smoking and second-hand smoke exposure with greater insulin resistance, an adverse lipid profile and a less favourable androgen profile [171]. However, the certainty of evidence was very low, and the observational nature of the available studies precludes conclusions regarding the effects of smoking cessation on these outcomes. Studies specifically addressing alcohol consumption in women with PMOS are limited, with inconsistent findings [58].

Taken together, these findings indicate that lifestyle management in PMOS should extend beyond the selection of dietary and exercise interventions to consideration of the broader behavioural and psychological context in which they are implemented. While the evidence base differs substantially across individual lifestyle domains, factors such as psychological wellbeing, disordered eating, sleep, stress and other health-related behaviours may influence the appropriateness, feasibility and sustainability of lifestyle modification. Their assessment may therefore be important for individualizing management, even where evidence for direct effects on metabolic or reproductive outcomes remains limited.

## 7. Potential Role of Lifestyle in PMOS Prevention and Early Risk Modification

PMOS frequently becomes clinically apparent during adolescence or early adulthood, although distinguishing pathological features from normal pubertal development remains challenging. The 2023 International Evidence-Based Guideline therefore recognizes a group of adolescents who show features suggestive of PMOS but do not yet meet full diagnostic criteria as being “at risk”, recommending ongoing reassessment and lifelong health planning [26,176]. In adolescents and other individuals without overweight, particular emphasis is placed on maintaining a healthy lifestyle and preventing excess weight gain [26,176]. These recommendations primarily address early risk modification and long-term health rather than established primary prevention of PMOS.

Among potentially modifiable early-life factors, excess adiposity has the strongest evidence linking it to subsequent PMOS. In a population-based cohort of 65,665 girls, childhood overweight between 7 and 13 years of age was associated with an approximately threefold higher subsequent risk of PMOS, with persistent overweight conferring greater risk than overweight at only one time point [177]. Consistent findings were reported in the Childhood Determinants of Adult Health and Bogalusa Heart Study cohorts, in which greater childhood adiposity was associated with menstrual irregularity and later PMOS, although associations varied according to population characteristics [178]. Particularly relevant evidence comes from the meta-analysis and Mendelian randomization study by Dobbie et al. [179]. In this analysis, each standard-deviation increase in genetically predicted BMI was associated with higher odds of PMOS (OR 2.76, 95% CI 2.27–3.35), while childhood body size remained independently associated with PMOS after adjustment for adult body size (OR 2.56, 95% CI 1.57–4.20). Genetically predicted fasting insulin was also strongly associated with PMOS risk. Although the observational component of this analysis was limited by cross-sectional and retrospective studies, the genetic evidence strengthens the hypothesis that excess adiposity and related metabolic dysfunction may contribute to PMOS development rather than merely coexist with established disease.

Evidence concerning specific dietary and physical-activity behaviours is less definitive. Cross-sectional studies in adolescents with established PCOS have reported higher fat intake, more frequent consumption of medium- or high-glycaemic-index foods and lower physical activity compared with unaffected peers; however, reverse causation and residual confounding cannot be excluded [180]. Similarly, a matched case–control study found higher sugar intake, BMI and physical inactivity to be associated with PCOS, whereas higher fibre and protein intake showed inverse associations [181]. A recent cross-sectional study of female university students similarly associated low physical activity, short sleep, frequent fast-food consumption, high stress, and overweight or obesity with higher odds of PMOS [182]. More compelling evidence is beginning to emerge from prospective cohorts. In Project Viva, a higher BMI z-score at a mean age of 13.1 years predicted PMOS in later adolescence (OR 1.72, 95% CI 1.15–2.56). This association was strongest among girls in the lowest tertile of objectively measured moderate-to-vigorous physical activity and weaker among more active participants, although the formal interaction test was not statistically significant [183]. These findings suggest that physical activity may attenuate adiposity-related risk.

Other lifestyle factors may also be relevant to early PMOS risk trajectories. A recent longitudinal analysis from the Raine Study followed sleep behaviours from childhood to adolescence and found that girls diagnosed with PCOS at age 14 had progressively more sleep difficulties during childhood; reported trouble sleeping at age 10 was associated with almost threefold higher odds of subsequent PCOS, while sleeping less than peers was associated with approximately 2.5-fold higher odds [184]. Beyond PMOS-specific risk, nutritional and lifestyle factors have also been examined in relation to menstrual health in young women. For example, Andreoli et al. [185] recently examined apparently healthy women aged 19–30 years and found only limited associations between diet, body composition, physical activity and menstrual regularity; the cross-sectional design, small sample and absence of detailed endocrine screening led the authors to regard their observations as hypothesis-generating.

Overall, current evidence supports early lifestyle-based risk modification, particularly through the prevention of excess weight gain and promotion of physical activity, while direct evidence for primary prevention of PMOS remains limited.

## 8. Conclusions

**Figure 1:**
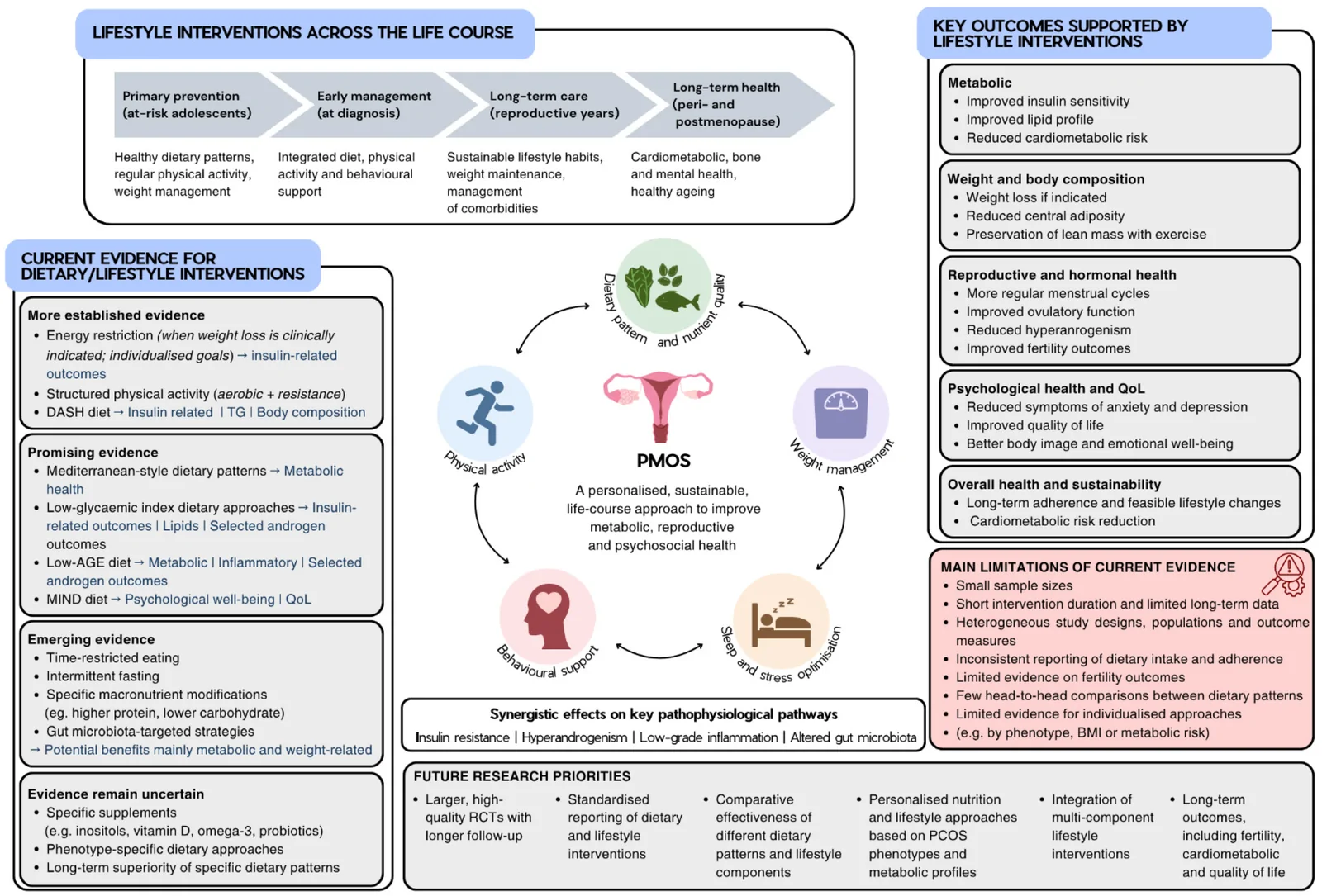
Evidence-based framework for the prevention and management of polyendocrine metabolic ovarian syndrome (PMOS). The figure summarizes current evidence on early risk modification, nutritional and lifestyle approaches, their predominant clinical outcomes, and the main limitations of the available evidence.

As summarized in Figure 1, the evidence reviewed in this article indicates that lifestyle intervention remains the cornerstone of PMOS management and may also play an important role in early risk modification. Diet, physical activity and broader behavioural and lifestyle support represent complementary components of PMOS management, addressing metabolic, reproductive, psychological and long-term cardiometabolic health. Rather than identifying a single optimal dietary model, current findings support individualized lifestyle strategies that address the predominant clinical and metabolic disturbances while remaining feasible and sustainable over time.

Among dietary strategies, multiple approaches appear effective in improving anthropometric, metabolic and reproductive outcomes. The choice of dietary strategy should be individualized according to clinical characteristics, reproductive and other treatment goals, patient preferences, and long-term feasibility. Exercise provides the strongest evidence among non-dietary interventions, whereas behavioural, psychological and mind–body approaches show potential complementary benefits, although their evidence base remains more limited. Tobacco exposure represents an additional modifiable health factor relevant to reproductive and cardiometabolic health, while PMOS-specific research on alcohol consumption remains limited. Sleep- and stress-focused interventions remain insufficiently studied. Dietary supplementation may provide additional benefits in selected women but should be regarded as complementary to, rather than a substitute for, lifestyle management. Pharmacological and, in selected cases, surgical treatment remain important adjuncts and should be individualized according to the predominant therapeutic goal and clinical indication.

Despite substantial progress, the current evidence base remains limited by considerable heterogeneity in diagnostic criteria, participant characteristics, intervention intensity, duration, dietary definitions and outcome measures. This heterogeneity complicates direct comparisons between studies and limits the generalizability of their findings. Most trials are relatively short, making it difficult to determine the long-term sustainability of observed benefits and to identify the interventions that are most effective across different patient populations and clinical presentations.

Future research should move beyond comparisons of individual diets and evaluate integrated, individualized lifestyle strategies for PMOS. Well-designed, adequately powered randomized trials with longer follow-up are needed to determine which combinations of dietary, physical activity, behavioural and adjunctive interventions are most effective across different clinical characteristics and life stages. Greater standardization of intervention definitions and outcome measures, together with longer follow-up, will also be important for translating the growing evidence base into more precise and clinically applicable lifestyle recommendations.

## Figures and Tables

**Table 1:** Summary of pharmacological and surgical management of PMOS.

Therapeutic Goal	Intervention	Mechanism of Action	Main Indication	Key Clinical Considerations	Position in Management
Menstrual cycle regulation and reduction in hyperandrogenic symptoms	Combined oral contraceptive pills (COCPs)	Suppression of pulsatile GnRH secretion reduces gonadotropin, particularly LH, secretion and consequently decreases ovarian androgen synthesis. The estrogen component also increases hepatic SHBG production, reducing circulating free androgen concentrations [28,51]	Menstrual irregularity, acne, hirsutism; contraception when pregnancy is not desired [26]	Reduce ovarian androgen production and increase SHBG; consider contraindications and thromboembolic risk [27,28]	First-line pharmacological option [26]
Improvement of metabolic parameters and insulin sensitivity	Metformin	Reduces hepatic glucose production by inhibiting gluconeogenesis and increases peripheral insulin sensitivity, largely through AMPK activation, reducing hyperinsulinaemia and indirectly decreasing ovarian androgen production [29,32,52]	Insulin resistance, impaired glucose metabolism, obesity; may also support ovulation [26,30]	Gastrointestinal adverse effects; rare lactic acidosis [31]	Adjunct to lifestyle intervention; may also support ovulation induction [26]
Weight reduction and metabolic improvement	GLP-1 receptor agonists	Activation of GLP-1 receptors increases satiety, delays gastric emptying and reduces appetite, resulting in weight loss; most benefits in PMOS appear to be mediated indirectly through weight reduction [53]	Women with obesity, particularly when lifestyle intervention is insufficient [26]		Adjunctive therapy [26,32]
Weight reduction and metabolic improvement	Orlistat/SGLT2 inhibitors	Orlistat inhibits gastric and pancreatic lipases, thereby reducing dietary fat absorption [33]. SGLT2 inhibitors promote urinary glucose excretion, contributing to improved glycaemic control, weight reduction and lower blood pressure [36].	Selected women with obesity or metabolic abnormalities [26]	Gastrointestinal adverse effects with orlistat [34]	Additional/alternative option [26]
Reduction in persistent clinical hyperandrogenism	Antiandrogens	Reduce androgen activity through androgen receptor blockade, inhibition of androgen production and/or inhibition of 5α-reductase-mediated conversion of testosterone to DHT [32]	Persistent hirsutism, acne or androgenic alopecia despite COCPs or when COCPs are contraindicated	Effective contraception required because of teratogenic potential [39]	Second-line [32,38]
Restoration of ovulation and achievement of pregnancy	Letrozole	Inhibits estrogen synthesis, reducing estrogen-mediated negative feedback on the hypothalamic–pituitary axis and increasing FSH secretion, which stimulates follicular development and ovulation [54]	Anovulatory infertility in women seeking pregnancy	Higher ovulation/live-birth efficacy than clomiphene in the evidence discussed [30]	First-line ovulation induction [26]
Restoration of ovulation and achievement of pregnancy	Clomiphene ± metformin	Clomiphene competitively blocks estrogen receptors in the hypothalamus, reducing negative estrogen feedback and increasing GnRH, FSH and LH secretion, thereby stimulating follicular development and ovulation [55].	Alternative induction strategy; especially with insulin resistance or clomiphene resistance [26,41]		Alternative/adjunctive therapy [26]
Restoration of ovulation and achievement of pregnancy	Gonadotropins	Exogenous FSH, or preparations containing FSH and LH, directly stimulate ovarian follicular growth and facilitate dominant follicle development and ovulation [56].	Persistent anovulatory infertility after failure of oral induction [26]	OHSS and multiple pregnancy risk; requires monitoring [43]	Further-line ovulation induction [26]
Achievement of pregnancy in treatment-resistant infertility	IVF		Failure of less invasive fertility treatment or additional infertility indications	Individualized stimulation due to higher OHSS risk [44]	Further-line fertility treatment
Restoration of ovulation in pharmacologically resistant infertility	LOD		Selected women with anovulatory infertility resistant to pharmacological therapy	Adhesions, possible reduction in ovarian reserve [47]	Selected surgical option
Restoration of ovulation in selected resistant infertility	TVOD		Potential alternative to LOD	Less invasive, but not established as standard of care [50]	Selected/non-standard option

AMPK, AMP-activated protein kinase; COCPs, combined oral contraceptive pills; DHT, dihydrotestosterone; FSH, follicle-stimulating hormone; GLP-1, glucagon-like peptide-1; GnRH, gonadotropin-releasing hormone; IVF, in vitro fertilization; LH, luteinizing hormone; LOD, laparoscopic ovarian drilling; OHSS, ovarian hyperstimulation syndrome; PMOS, polyendocrine metabolic ovarian syndrome; SGLT2, sodium-glucose co-transporter 2; SHBG, sex hormone-binding globulin; TVOD, transvaginal ovarian drilling.

**Table 4:** Effects of higher-protein dietary approaches on metabolic and reproductive outcomes in women with PMOS.

Study	Design/Sample	Intervention/Comparator	Duration	Key Outcomes	Clinical Interpretation
Mehrabani et al., 2012 [98]	Single-blind RCT; 60 women with overweight/obesity and PMOS	High-protein, low-GL hypocaloric diet vs. conventional hypocaloric diet	12 weeks	Similar weight loss; greater reductions in fasting insulin, HOMA-IR and hsCRP with the high-protein/low-GL diet; testosterone decreased with both diets	Suggests metabolic effects beyond weight loss, although the intervention combined higher protein intake with lower glycaemic load
Toscani et al., 2011 [101]	RCT; 18 women with PMOS (9 high-protein, 9 normal-protein)	High-protein vs. normal-protein diet	8 weeks	Similar reductions in weight, BMI, waist circumference, body fat and testosterone with both diets; no clear between-diet differences in metabolic outcomes	No additional short-term benefit of increasing protein content was demonstrated

BMI, body mass index; GL, glycaemic load; HOMA-IR, homeostasis model assessment of insulin resistance; hsCRP, high-sensitivity C-reactive protein; PMOS, polyendocrine metabolic ovarian syndrome; RCT, randomized controlled trial.

**Table 5:** Effects of dietary pattern interventions on metabolic, reproductive, and psychological outcomes in women with PMOS.

Study	Design/Sample	Diet Comparison	Duration	Main Effects in PMOS	Clinical Interpretation
Azadi-Yazdi et al., 2017 [103]	Randomized controlled trial; 60 overweight/obese women with PMOS	Energy-restricted DASH diet vs. energy-restricted control diet; both diets provided 50–55% carbohydrate, 15–20% protein, and 25–30% fat	3 months	DASH produced larger reductions in weight, BMI, fat mass, and androstenedione, and larger increases in SHBG and antioxidant capacity than control	A calorie-restricted DASH pattern improved both body composition and selected androgen/oxidative stress markers, supporting DASH as a strong whole-diet option in PMOS
Scannell et al., 2025 [104]	Pilot RCT; 26 women with PMOS and BMI ≥ 25 kg/m^2^	Ad libitum Mediterranean diet vs. healthy-eating control; no caloric restriction	12 weeks	Mediterranean diet intervention was feasible and acceptable, with greater dietary adherence than healthy-eating advice	Supports the feasibility of a Mediterranean dietary pattern in PMOS; the pilot design and small sample preclude conclusions regarding clinical efficacy
Kabiri et al., 2024 [105]	RCT; 52 women with PCOS, aged 18–45 years, randomized to intervention or control	Low-calorie MIND diet vs. control diet; MIND combines Mediterranean and DASH principles	8 weeks	Reduced depression and anxiety scores and improved PCOSQ emotion and body hair domains; most other metabolic, anthropometric, and hormonal outcomes did not differ significantly between groups	MIND is a promising food-quality model for psychological and quality-of-life outcomes in PMOS, but current evidence is still early and does not show broad short-term metabolic or hormonal benefit
Ozdemir et al., 2025 [106]	RCT; 44 phenotype-A overweight women with PMOS	Low-AGEs weight-loss diet vs. standard AGEs-containing weight-loss diet	12 weeks	Weight loss was similar, but low-AGEs diet improved fasting glucose more and reduced WHR, LDL-C, TNF-α, testosterone, FAI, and AMH, while increasing SHBG	Lowering dietary AGEs may add metabolic and hormonal benefit beyond weight loss alone
Wang et al., 2022 [107]	Randomized trial; 25 women with PMOS	High-fiber diet vs. high-fiber diet + acarbose	12 weeks	Both improved clinical phenotypes, inflammation, reproductive function, and brain–gut peptides; combination arm showed greater improvement in hyperandrogenism and insulin resistance	Fiber-rich modified diets appear promising, though the added drug means this is not a pure diet-only comparison

AGEs, advanced glycation end products; AMH, anti-Müllerian hormone; BMI, body mass index; DASH, Dietary Approaches to Stop Hypertension; FAI, free androgen index; LDL-C, low-density lipoprotein cholesterol; MIND, Mediterranean-DASH Intervention for Neurodegenerative Delay; PCOS, polycystic ovary syndrome; PMOS, polyendocrine metabolic ovarian syndrome; PCOSQ, Polycystic Ovary Syndrome Health-Related Quality of Life Questionnaire; RCT, randomized controlled trial; SHBG, sex hormone-binding globulin; TNF-α, tumor necrosis factor alpha; WHR, waist-to-hip ratio.

**Table 6:** Meta-Analyses and systematic reviews of dietary interventions in PMOS.

Diet/Approach	Review	Number of Studies	Key Pooled Findings	Evidence Certainty/Risk of Bias	Duration	Key Limitation
Multiple dietary approaches (network meta-analysis)	Juhász et al., 2024 [17]	19 RCTs; 727 participants	DASH ranked highest for HOMA-IR (SUCRA 80.4%) and fasting insulin (79.7%); low-calorie diet ranked highest for BMI (73.6%)	GRADE: low–high, depending on comparison	4–24 weeks	Few direct comparisons; small sample sizes and variable intervention duration
Multiple dietary approaches–insulin resistance and body composition	Shang et al., 2020 [81]	19 trials; 1193 participants	↓ HOMA-IR: MD −0.78 (95% CI −0.92 to −0.65; I^2^ = 24%); ↓ BMI: MD −1.01 kg/m^2^ (95% CI −1.38 to −0.64; I^2^ = 54%); subgroup analyses favoured DASH for insulin resistance and calorie-restricted diets for body composition	Cochrane RoB; some concerns, particularly attrition	4 weeks–1 year; mostly ≤12 weeks	Limited geographic diversity and predominantly short interventions
Multiple dietary approaches in women with BMI ≥ 25 kg/m^2^	Yang et al., 2024 [80]	9 RCTs; 559 participants	↓ weight (MD −3.29 kg), BMI (MD −3.49 kg/m^2^), HOMA-IR (MD −0.76), and LH (MD −0.83 IU/L); ↑ pregnancy (RR 2.51) and ovulation (RR 1.48)	Cochrane RoB; generally low risk for incomplete outcome data, but selective reporting and other bias were unclear	2–4 months	High heterogeneity for several outcomes; heterogeneous interventions and comparators; many diet-specific subgroup estimates based on single trials
Low-carbohydrate diets	Zhang et al., 2019 [87]	8 RCTs; 327 participants	↓ BMI: SMD −1.04 (95% CI −1.38 to −0.70; I^2^ = 0%); ↓ HOMA-IR: SMD −0.66 (95% CI −1.01 to −0.30; I^2^ = 30%); ↑ SHBG: MD 6.02 (95% CI 3.55 to 8.48; I^2^ = 0%); total testosterone NS overall	Cochrane RoB; predominantly low or unclear risk, with limited blinding	1–24 weeks	Small number of RCTs; high heterogeneity for testosterone
Low-GI diets	Kazemi et al., 2021 [88]	10 RCTs; 403 participants	↓ HOMA-IR: WMD −0.78 (95% CI −1.20 to −0.37; I^2^ = 86.6%); ↓ triglycerides: WMD −14.85 mg/dL (95% CI −28.75 to −0.95; I^2^ = 31%); ↓ total testosterone: WMD −0.21 nmol/L (95% CI −0.32 to −0.09; I^2^ = 8.6%)	Cochrane RoB; mostly low or unclear risk	8 weeks–6 months	High heterogeneity for HOMA-IR; HOMA-IR benefit was evident mainly in energy-restricted trials
High-fibre and low-GI/GL diets	Zhang et al., 2025 [99]	13 RCTs	**High-fibre:** ↓ HOMA-IR: SMD −0.43 (95% CI −0.83 to −0.02; I^2^ = 56%); ↑ SHBG: SMD 0.70 (95% CI 0.41 to 0.99; I^2^ = 0%). **Low-GI:** ↓ HOMA-IR: SMD −0.28 (95% CI −0.55 to −0.02; I^2^ = 46%); ↓ FAI: SMD −0.36 (95% CI −0.63 to −0.10; I^2^ = 48%)	Cochrane RoB; overall moderate quality	6 h–6 months	Heterogeneity across interventions and outcomes; limited evidence for low-GL diets
Ketogenic diet	Arsenaki et al., 2026 [100]	15 studies; 10 included in meta-analysis	Compared with other diets: ↓ BMI (MD −1.65 kg/m^2^), body weight (MD −4.98 kg), HOMA-IR (MD −1.71), and LH (MD −1.68 IU/L); androgen and lipid outcomes were inconsistent	GRADE; 2/5 RCTs low risk of bias, 3/5 with some concerns or high risk; observational evidence also included	Up to 8 months	Mixed study designs; substantial heterogeneity for several outcomes; limited long-term evidence
High-protein diets	Wang et al., 2024 [102]	8 RCTs; 300 participants	↓ fasting insulin: WMD −2.69 μIU/mL (95% CI −3.81 to −1.57; I^2^ = 46%); ↓ HOMA-IR: WMD −0.41 (95% CI −0.78 to −0.03; I^2^ = 96%)	Cochrane RoB; variable risk of bias across trials	4–16 weeks	Short interventions; very high heterogeneity for HOMA-IR, with the pooled effect sensitive to individual studies
Time-restricted eating (TRE)	Hamsho et al., 2026 [111]	4 RCTs; 216 participants	↓ HOMA-IR: MD −0.58 (95% CI −0.87 to −0.30; I^2^ = 0%); ↑ QUICKI: MD 0.08 (95% CI 0.04 to 0.13; I^2^ = 22%); ↑ HDL-C: MD 1.97 mg/dL (95% CI 0.96 to 2.99; I^2^ = 0%)	Overall certainty of evidence limited	2–6 months	Only 4 RCTs with modest sample sizes; heterogeneous TRE protocols and comparators
Dietary interventions–reproductive outcomes	Shang et al., 2021 [71]	20 RCTs; 1113 participants	↑ clinical pregnancy: RR 2.87 (95% CI 1.99–4.13; I^2^ = 0%); ↑ ovulation: RR 1.30 (95% CI 1.10–1.53; I^2^ = 0%); subgroup analyses favoured low-carbohydrate diets for reproductive outcomes, whereas calorie restriction was more consistently associated with improvements in hyperandrogenism	Cochrane RoB; mixed risk of bias across trials	Mostly 3–6 months; range up to 1 year	Heterogeneous dietary interventions; several reproductive outcomes based on relatively few trials

BMI, body mass index; CI, confidence interval; DASH, Dietary Approaches to Stop Hypertension; FAI, free androgen index; GI, glycaemic index; GL, glycaemic load; GRADE, Grading of Recommendations Assessment, Development and Evaluation; HDL-C, high-density lipoprotein cholesterol; HOMA-IR, homeostasis model assessment of insulin resistance; I^2^, inconsistency statistic; LH, luteinizing hormone; MD, mean difference; NS, not significant; PMOS, polyendocrine metabolic ovarian syndrome; QUICKI, quantitative insulin sensitivity check index; RCT, randomized controlled trial; RoB, risk of bias; RR, risk ratio; SHBG, sex hormone-binding globulin; SMD, standardized mean difference; SUCRA, surface under the cumulative ranking curve; WMD, weighted mean difference. ↑ Increase; ↓ decrease.

**Table 7:** Characteristics and main findings of intervention studies evaluating dietary supplements in women with PMOS.

Study	Design/Sample	Dose/Duration	Main Findings	Key Limitation
**Inositols**				
Costantino et al., 2009 [131]	Double-blind placebo-controlled RCT; 42 women with PMOS	Myo-inositol 4 g/day + folic acid 400 μg/day vs. folic acid 400 μg/day; 6–8 weeks	↓ total and free testosterone, TG and BP; improved insulin sensitivity and ovulatory function	limited reporting of randomization procedures
Ravn et al., 2022 [132]	Open-label RCT; 45 women with PMOS	Myo-inositol 4 g/day vs. metformin 2 g/day; 6 months	Similar improvement in cycle length; no significant improvement in HOMA-IR with MI; metformin produced greater reductions in weight and fasting glucose; fewer adverse effects with MI	Small sample, high attrition and open-label design
Gul et al., 2025 [133]	Prospective clinical trial; 60 participants	MI + DCI 40:1 vs. metformin, 12 weeks	Both groups improved HOMA-IR, SHBG, ovarian volume, menstrual regularity, BMI, QoL, and stress scores; metformin was slightly better in some parameters, while MI + DCI may differ by phenotype	Small study and phenotype-specific claims remain exploratory.
**Vitamin D**				
Wen et al., 2024 [134]	RCT; 60 women with PMOS and vitamin D deficiency/insufficiency; 57 completed	2000 IU/day vs. control; 12 weeks	↓ BMI, WHR, insulin, HOMA-IR, TG, TC and LDL-C; benefits were more evident in women with obesity or IR	Small sample; subgroup analyses included very small groups
Trummer et al., 2018 [135]	Double-blind placebo-controlled RCT; 180 randomized; 123 completed	20,000 IU/week vs. placebo; 24 weeks	↑ 25(OH)D; no effect on primary glucose AUC outcome; ↓ 60 min OGTT glucose as a secondary outcome	High attrition; overall metabolic effects were limited
Javed et al., 2019 [136]	Double-blind placebo-controlled RCT; 40 women with PMOS	3200 IU/day vs. placebo; 3 months	↓ ALT; borderline improvement in HOMA-IR; no clear effects on other metabolic or hormonal outcomes	Small exploratory trial
**Omega-3 fatty acids**				
Phelan et al., 2011 [137]	Randomized, double-blind, placebo-controlled crossover trial; 22 women with PMOS completed	LC n-3 PUFA 2.4 g/day (EPA + DHA 1.9 g/day; EPA:DHA 1.49:1) vs. olive-oil placebo; 6 weeks per treatment, with 6-week washout	↓ bioavailable testosterone; no significant changes in total testosterone, DHEAS, SHBG, fasting/postprandial glucose, insulin or TG	Small sample and short intervention
Cussons et al., 2009 [138]	Randomized placebo-controlled crossover trial; 25 women with PMOS	Fish oil 4 g/day (EPA 675 mg + DHA 1400 mg/day) vs. olive-oil placebo; 8 weeks	↓ hepatic fat and TG; ↓ systolic and diastolic BP; reduction in hepatic fat particularly evident in women with hepatic steatosis	Small sample and short intervention
**NAC**				
Oner & Muderris, 2011 [139]	Prospective randomized trial; 100 women with PMOS randomized; 75 completed	NAC 1.8 g/day vs. metformin 1.5 g/day; 24 weeks	Both treatments ↓ BMI, fasting insulin, HOMA-IR, free testosterone and menstrual irregularity; broadly comparable effects between treatments	High and unequal attrition, particularly in the metformin group; active-comparator trial without placebo
Fang et al., 2024 [140]	Pragmatic randomized controlled clinical study; 230 women	1.8 g/day, up to 3 ovulation induction cycles	NAC reduced uFSH dose and duration and increased clinical pregnancy rates per cycle and cumulative pregnancy rates per patient during sequential letrozole/uFSH induction	Adjunct setting in ovulation induction rather than stand-alone supplementation.

ALT, alanine aminotransferase; AUC, area under the curve; BMI, body mass index; BP, blood pressure; DCI, D-chiro-inositol; DHA, docosahexaenoic acid; DHEAS, dehydroepiandrosterone sulfate; EPA, eicosapentaenoic acid; HOMA-IR, homeostasis model assessment of insulin resistance; IR, insulin resistance; IU, international units; LC n-3 PUFA, long-chain n-3 polyunsaturated fatty acids; LDL-C, low-density lipoprotein cholesterol; MI, myo-inositol; NAC, N-acetylcysteine; OGTT, oral glucose tolerance test; PMOS, polyendocrine metabolic ovarian syndrome; QoL, quality of life; RCT, randomized controlled trial; SHBG, sex hormone-binding globulin; TC, total cholesterol; TG, triglycerides; uFSH, urinary follicle-stimulating hormone; WHR, waist-to-hip ratio; 25(OH)D, 25-hydroxyvitamin D. ↑ Increase; ↓ decrease.

**Table 8:** Summary of meta-analyses and systematic reviews evaluating dietary supplements in women with PMOS.

Supplement	Review	Number of Studies	Key Pooled Findings	Evidence Certainty/Risk of Bias	Dosing/Duration	Key Limitation
**Inositols**	Fitz et al., 2024 [141]	30 trials; 2230 participants; 19 pooled	DCI vs. placebo: ↓ free testosterone: MD −0.46 (95% CI −0.76 to −0.15); ↑ ovulation: OR 11.50 (3.40–38.91); effects on several other outcomes inconsistent	Predominantly low to very low certainty; selected MI vs. metformin outcomes moderate	Various MI, DCI and combined MI/DCI regimens; generally ≥6 weeks to 6 months	Very small samples and imprecision; heterogeneous inositol formulations, comparators and co-interventions
	Unfer et al., 2017 [142]	9 RCTs; 496 participants	↓ fasting insulin: SMD −1.02 (95% CI −1.79 to −0.25; I^2^ = 90.6%); ↓ HOMA-IR: SMD −0.59 (−1.15 to −0.03; I^2^ = 84.1%); ↑ SHBG only in 24-week subgroup	Methodological quality variable; incomplete reporting in several domains	MI 1.1–4 g/day; 12–24 weeks	Very high heterogeneity for insulin and HOMA-IR; variable PMOS criteria, MI doses and treatment durations
**Vitamin D**	Zhang et al., 2023 [143]	13 RCTs; 840 participants	↑ endometrial thickness: MD 1.78 (95% CI 0.49–3.06); ↓ hs-CRP: MD −0.54 (−1.00 to −0.08); ↓ TC: MD −12.00 (−18.36 to −5.56); ↓ total testosterone: MD −0.17 (−0.29 to −0.05)	Cochrane RoB; methodological quality varied across trials	Highly variable regimens (daily, weekly, biweekly or bolus dosing); 8–24 weeks	Small RCTs and substantial variation in supplementation regimens and outcome assessment
	Yu et al., 2025 [144]	13 RCTs; 691 participants	↓ FBG: MD −2.91 mg/dL (95% CI −4.78 to −1.04; I^2^ = 27%); ↓ insulin: MD −1.98 µIU/mL (−3.32 to −0.64; I^2^ = 38%); ↓ TG: MD −11.01 mg/dL (−16.42 to −5.61; I^2^ = 12%); ↓ TC: MD −11.69 mg/dL (−15.56 to −7.82; I^2^ = 14%)	GRADE: low for FBG, insulin and TG; moderate for TC	Variable vitamin D3 regimens (daily, weekly or biweekly dosing); 8–24 weeks	Limited number and sample size of RCTs; optimal dose and long-term effects remain uncertain
**Omega-3 fatty acids**	Yuan et al., 2021 [145]	10 RCTs; 610 participants	↓ CRP: WMD −8.97 (95% CI −17.66 to −0.28; I^2^ = 99%); ↓ MDA: WMD −0.40 (−0.56 to −0.25; I^2^ = 42%); ↓ total testosterone: WMD −0.11 (−0.18 to −0.04; I^2^ = 73%); ↑ SHBG: WMD 0.68 (0.06–1.31; I^2^ = 0%)	Cochrane RoB; randomization method unclear in 5/10 and allocation concealment unclear in 8/10 RCTs	n-3 PUFA 1000–3500 mg/day; 6–12 weeks	Few RCTs and marked outcome-specific heterogeneity, particularly for CRP and testosterone
**Microbiota-targeted supplements**	Maddirevula et al., 2025 [127]	17 controlled trials; 1214 participants	↓ total testosterone: MD −0.19 ng/mL (95% CI −0.30 to −0.08); ↓ LH/FSH ratio: SMD −0.46 (−0.66 to −0.26); ↓ fasting insulin: MD −2.4 µIU/mL (−3.9 to −0.9); ↓ HOMA-IR: MD −0.49 (−0.78 to −0.19)	GRADE: moderate for insulin-related outcomes; low for sex-hormone outcomes; 8 trials low RoB, 6 some concerns, 3 high RoB	Probiotics, synbiotics and postbiotic/metabolite interventions; ≥8 weeks	Heterogeneous interventions; small samples and relatively short duration; limited evidence for long-term and clinically relevant reproductive outcomes
**NAC**	Thakker et al., 2015 [146]	8 RCTs; 910 participants	vs placebo: ↑ live birth: OR 3.00 (95% CI 1.05–8.60); ↑ pregnancy: OR 3.58 (2.05–6.25); ↑ ovulation: OR 3.13 (1.54–6.36); vs. metformin: lower pregnancy and ovulation rates	High selection/performance/attrition risk in 2 RCTs; high reporting-bias risk in 4	NAC regimens varied across trials; study duration 2–12 months	Live-birth evidence based on a single trial; 5/8 RCTs involved clomiphene-resistant PMOS; substantial risk of bias in several trials
	Liu et al., 2023 [147]	11 RCTs; 869 participants	↓ FBG vs. metformin: SMD −0.23 (95% CI −0.43 to −0.04); ↓ FBG vs. placebo: SMD −0.54 (−1.03 to −0.05); ↓ TC vs. placebo: SMD −0.74 (−1.37 to −0.12); no clear benefit for most other metabolic outcomes	Cochrane RoB; randomization/allocation concealment incompletely reported in most trials	NAC dosing varied across included-study reports; 6–24 weeks	Predominantly Asian studies and strong comparator imbalance: most trials used metformin, very few placebo

CI, confidence interval; CRP, C-reactive protein; DCI, D-chiro-inositol; FBG, fasting blood glucose; FSH, follicle-stimulating hormone; GRADE, Grading of Recommendations Assessment, Development and Evaluation; HOMA-IR, homeostasis model assessment of insulin resistance; hs-CRP, high-sensitivity C-reactive protein; IU, international units; LH, luteinizing hormone; MDA, malondialdehyde; MD, mean difference; MI, myo-inositol; NAC, N-acetylcysteine; OR, odds ratio; PMOS, polyendocrine metabolic ovarian syndrome; RCT, randomized controlled trial; RoB, risk of bias; SHBG, sex hormone-binding globulin; SMD, standardized mean difference; TC, total cholesterol; TG, triglycerides; WMD, weighted mean difference. ↑ Increase; ↓ decrease.

**Table 9:** Studies on non-dietary lifestyle factors in PMOS.

Study	Design/Sample	Lifestyle Factor/Intervention	Main Findings	Key Limitation
Woodward et al., 2022 [164]	Randomized controlled feasibility trial; 36 women	Supervised aerobic exercise vs. lifestyle PA intervention targeting sedentary behaviour vs. control; 12 weeks	Lifestyle PA showed higher adherence than supervised exercise (100% vs. 53%); both intervention groups lost weight, while supervised exercise reduced oxidized LDL	Feasibility study; not powered to establish efficacy
Cao et al., 2023 [165]	Cross-sectional; 283 infertile women with PMOS	Sedentary behaviour	Sitting > 7 h/day was associated with poorer total and domain-specific HRQoL independently of PA, anxiety and depression	Cross-sectional design; restricted to women with infertility
Moran et al., 2013 [65]	Prospective cohort analysis; 409 women with PMOS and 7057 controls	Diet, PA and sedentary behaviour	PMOS status, higher energy intake and dietary GI, and lower PA were independently associated with higher BMI; women with PMOS also reported more sitting time	PMOS status and lifestyle behaviours were self-reported
Kite et al., 2025 [161]	UK Biobank observational analysis; 319 women with PMOS, 638 age-matched and 638 age- and BMI-matched controls	PA and sedentary behaviour in women aged >40 years	Women with PMOS reported less vigorous PA and more screen/sedentary time; lower PA and greater sedentary behaviour were associated with less favourable health profiles across groups	Observational design; causal relationships cannot be established
Peebles et al., 2025 [162]	Large digital cohort; 11,022 women with PMOS and 7152 with possible PMOS	Exercise, sleep and stress-related lifestyle characteristics	PMOS and possible PMOS were associated with less favourable exercise, sleep and stress profiles; abnormal sleep duration and sleep-apnoea-related outcomes were more frequent in PMOS	PMOS status and lifestyle characteristics largely self-reported
Vela et al., 2026 [11]	Community-based cross-sectional study; 1574 participants	Depression, anxiety, sleep and PA according to PMOS status and BMI	PMOS was associated with greater depression, anxiety and insomnia burden; elevated sleep-apnoea risk was particularly evident in higher-BMI groups	Cross-sectional design; self-reported PMOS and BMI
Verde et al., 2026 [166]	Case–control study; 112 women with PMOS and 112 age- and BMI-matched controls	Sleep quality, body composition, dietary intake and Mediterranean diet adherence	Poorer sleep quality in PMOS was associated with greater fat mass, less favourable metabolic and inflammatory profiles and lower Mediterranean diet adherence; Mediterranean diet adherence was the strongest overall predictor of sleep quality	Observational design; associations do not establish causality
Nidhi et al., 2012 [167]; Nidhi et al., 2013 [168]	Randomized active-controlled trial; 90 adolescent girls with PMOS	Holistic yoga vs. conventional physical exercise; 1 h/day for 12 weeks	Yoga resulted in greater improvements in fasting glucose, fasting insulin and HOMA-IR, and in selected endocrine outcomes including AMH, LH and testosterone; menstrual frequency also improved	Adolescent population from a single residential setting; short intervention

AMH, anti-Müllerian hormone; BMI, body mass index; GI, glycemic index; HOMA-IR, homeostasis model assessment of insulin resistance; HRQoL, health-related quality of life; LDL, low-density lipoprotein; LH, luteinizing hormone; PA, physical activity; PMOS, polyendocrine metabolic ovarian syndrome.

**Table 10:** Meta-analyses and systematic reviews of non-dietary or mixed lifestyle interventions in PMOS.

Approach	Review	Number of Studies	Key Pooled/Overall Findings	Evidence Certainty/Risk of Bias	Duration	Key Limitation
Physical activity behaviour-observational evidence	Kazemi et al., 2022 [163]	54 studies; 39,471 participants	Women with PCOS had lower total PA than controls; differences in vigorous PA and sedentary behaviour were less consistent	Overall evidence limited by methodological quality and heterogeneity of behavioural assessment	Cross-sectional/observational studies	Predominantly self-reported PA; observational evidence cannot establish direction of association
Exercise-fitness, adiposity and insulin resistance	Patten et al., 2020 [159]	33 studies; 19 meta-analysed; 777 women	Vigorous exercise: ↑ VO_2_peak 24.2% (90% CL 18.5–30.1); ↓ HOMA-IR 36.2% (90% CL 9.0–55.3); ↓ waist circumference 4.2% (90% CL 2.3–6.0)	Variable study quality; evidence strongest for vigorous-intensity exercise	6–26 weeks	Heterogeneous exercise protocols; relatively short interventions
Exercise-cardiometabolic outcomes	Breyley-Smith et al., 2022 [160]	18 studies; 593 participants	↑ cardiorespiratory fitness: WMD 4.00 mL/kg/min (95% CI 2.61–5.40); ↓ waist circumference: WMD −1.48 cm (95% CI −2.35 to −0.62); no clear pooled effects on glucose, HOMA-IR or lipid profile	Risk of bias varied across trials	≥4 weeks	Most participants had overweight/obesity and relatively normal baseline cardiometabolic markers
Aerobic and resistance exercise	Cavalcante et al., 2025 [169]	10 RCTs; 382 women	Aerobic exercise reduced fasting insulin, total cholesterol and triglycerides in pooled analyses; resistance exercise showed favourable changes in body composition, but evidence was sparse	RCT evidence; methodological quality and intervention protocols varied	12–24 weeks	Meta-analysis was driven predominantly by aerobic exercise; limited evidence for resistance training
Exercise-androgen outcomes	Atmaca et al., 2026 [170]	23 studies; 12 studies/563 participants for total testosterone	No overall effect of exercise on total testosterone, DHEA-S or SHBG; aerobic exercise subgroup ↓ total testosterone: MD −33.96 (95% CI −46.10 to −21.82; I^2^ = 0%)	RoB varied; sensitivity analyses did not materially change the main findings	Mostly 8–24 weeks	Substantial heterogeneity for overall testosterone effect; few studies within exercise-modality subgroups
Smoking and second-hand smoke exposure	Yan et al., 2026 [171]	9 studies; 3053 women	↑ HOMA-IR: SMD 0.16 (95% CI 0.05–0.27); ↑ TG: SMD 0.25 (95% CI 0.08–0.42); ↑ total testosterone: SMD 0.21 (95% CI 0.12–0.29); ↓ SHBG: SMD −0.19 (95% CI −0.74 to −0.08)	Very low (GRADE)	Observational studies	Observational evidence; active and passive exposure combined in primary analyses
Yoga-mind–body intervention	Verma et al., 2023 [172]	9 studies (11 publications); 515 participants; 2 RCTs included in meta-analysis	↓ clinical hyperandrogenism: MD −0.70 (95% CI −1.15 to −0.26); ↓ HOMA-IR: MD −0.86 (95% CI −1.29 to −0.43; I^2^ = 0%); ↓ menstrual irregularity: MD −0.41 (95% CI −0.74 to −0.08; one RCT)	Low certainty (GRADE)	6 weeks–6 months	Only 2 RCTs contributed to quantitative analyses; small samples and heterogeneous yoga interventions
Mixed lifestyle interventions–reproductive and psychological outcomes	Mohamed et al., 2025 [173]	24 studies; 1373 women	Lifestyle interventions incorporating diet, exercise and/or behavioural strategies were generally associated with improvements in reproductive, psychological and QoL outcomes	Systematic review without quantitative pooling because of substantial heterogeneity	8 weeks-12 months	Mixed intervention components and outcomes prevent attribution of effects to specific non-dietary strategies

CI, confidence interval; CL, confidence limits; DHEA-S, dehydroepiandrosterone sulfate; GRADE, Grading of Recommendations Assessment, Development and Evaluation; HOMA-IR, homeostasis model assessment of insulin resistance; I^2^, inconsistency statistic; MD, mean difference; PA, physical activity; PCOS, polycystic ovary syndrome; PMOS, polyendocrine metabolic ovarian syndrome; QoL, quality of life; RCT, randomized controlled trial; RoB, risk of bias; SHBG, sex hormone-binding globulin; SMD, standardized mean difference; TG, triglycerides; VO_2_peak, peak oxygen uptake; WMD, weighted mean difference. ↑ Increase; ↓ decrease.

## Data Availability

No new data were created or analyzed in this study. Data sharing is not applicable to this article.
